# Impact of Preeclampsia Duration on Long-Term Cardiovascular Disease Risk

**DOI:** 10.1161/HYPERTENSIONAHA.125.25054

**Published:** 2025-11-18

**Authors:** Nina Keitaanpää, Jaakko S. Tyrmi, Elli Toivonen, Heini Huhtala, Anni Kivelä, Seppo Heinonen, Tiina Jääskeläinen, Hannele Laivuori

**Affiliations:** Center for Child, Adolescent, and Maternal Health Research, Faculty of Medicine and Health Technology (N.K., J.S.T., E.T., H.L.), Tampere University, Finland.; Faculty of Social Sciences (H.H.), Tampere University, Finland.; Center for Life Course Health Research, Faculty of Medicine, University of Oulu, Finland (J.S.T.).; Department of Obstetrics and Gynecology, Tampere University Hospital, the Wellbeing Services County of Pirkanmaa, Finland (E.T., H.L.).; Medical and Clinical Genetics, University of Helsinki and Helsinki University Hospital, Finland (A.K., T.J., H.L.).; Department of Obstetrics and Gynecology, Helsinki University Hospital and University of Helsinki, Finland (S.H.).; Department of Food and Nutrition, University of Helsinki, Finland (T.J.).; Institute for Molecular Medicine Finland, Helsinki Institute of Life Science, HiLIFE, University of Helsinki, Finland (H.L.).

**Keywords:** cardiovascular disease, gestational age, hypertension, preeclampsia, pregnancy complications, risk factors

## Abstract

**BACKGROUND:**

Preeclampsia is associated with increased risk of subsequent maternal cardiovascular diseases (CVD) compared with normotensive pregnancies. We examined whether a longer duration between preeclampsia diagnosis and delivery is associated with a higher CVD risk before age 55.

**METHODS:**

Nationwide health registry data from 2 Finnish cohorts were used: FINNPEC (n=1139) and FinnGen (n=3603). Exposure was the duration from preeclampsia diagnosis to delivery in days. Outcome was a composite CVD, including new-onset hypertensive diseases, ischemic heart diseases, cerebral/precerebral arterial diseases, and peripheral artery diseases diagnosed before age 55. Cox proportional hazards models were used to estimate the risk of composite CVD for each day between preeclampsia diagnosis and delivery.

**RESULTS:**

Median follow-up time after delivery was 12.2 years (interquartile range, 11.0–15.5) in FINNPEC and 17.1 years (interquartile range, 12.5–22.2) in FinnGen. A longer preeclampsia duration was associated with elevated CVD risk on a daily basis, with a hazard ratio of 1.02 (95% CI, 1.00–1.04); *P*=0.020 in FINNPEC and hazard ratio of 1.01 (95% CI, 1.00–1.02); *P*<0.001 in FinnGen. In FINNPEC, the association between preeclampsia duration and CVD remained significant after adjustment for body mass index, maternal age at delivery, small for gestational age infant, and smoking (adjusted hazard ratio, 1.02 [95% CI, 1.00–1.05]; *P*=0.022). In FinnGen, the association was observed after adjustment for early-onset preeclampsia, pregestational and gestational diabetes, primiparity, and age at delivery (adjusted hazard ratio, 1.01 [95% CI, 1.00–1.01]; *P*=0.012).

**CONCLUSIONS:**

Long-term CVD risk before age 55 is estimated to increase 1% to 2% per day from preeclampsia diagnosis to delivery. Women with preeclampsia might benefit from early delivery.

**NOVELTY AND RELEVANCE**

**What Is New?**

To the best of our knowledge, the impact of the duration from preeclampsia diagnosis to delivery on maternal cardiovascular morbidity has not been studied before with follow-up periods as long as in our study.

**What Is Relevant?**

A longer duration between preeclampsia diagnosis and delivery is associated with an increased long-term risk of cardiovascular disease. These findings suggest that prolonged exposure to endothelial dysfunction during preeclampsia may affect future cardiovascular morbidity.

**Clinical/Pathophysiological Implications?**

Early delivery in preeclamptic pregnancies may improve women’s long-term cardiovascular health. The duration of preeclampsia should be considered when assessing long-term cardiovascular risk and planning preventive care.

Preeclampsia is a multisystem pregnancy disorder affecting 3% to 5% of pregnancies^1,2^ and one of the major causes of maternal and perinatal morbidity and mortality worldwide.^3^ Endothelial dysfunction caused by placental malperfusion leads to the classical clinical features of preeclampsia, including maternal hypertension and proteinuria developing after 20 weeks of gestation (wg).^4,5^ However, the clinical presentation of preeclampsia is highly variable.^4–6^

Preeclampsia and other hypertensive pregnancy disorders (HDPs) are associated with a ≈2- to 4-fold long-term risk of maternal cardiovascular diseases (CVDs), including chronic hypertension, compared with normotensive pregnancies.^7–11^ Endothelial dysfunction, either induced or exacerbated by preeclampsia, is potentially an important pathophysiological mechanism in CVD development.^12^ Women with early-onset preeclampsia, requiring delivery or diagnosis before 34^0/7^ wg, are at particularly high risk of subsequent CVDs, compared with those with late-onset preeclampsia.^11,13^ Pregnancy prolongation in preeclampsia pregnancies increases short-term maternal morbidity but improves perinatal outcomes.^14,15^ However, evidence is conflicting on whether preeclampsia duration (latency from diagnosis to delivery) is associated with long-term maternal cardiovascular morbidity in follow-up periods from 6 months to 5.2 years.^16–20^ Identifying women with the highest CVD risk would aid in targeting CVD prevention.

The main objective of this study was to examine whether delaying delivery after preeclampsia diagnosis is associated with increased risk of maternal CVDs before the age of 55. The secondary objective was to identify other high-risk subgroups who could benefit from postpartum CVD prevention. In this study, a significantly longer follow-up after delivery could be provided compared with previous studies.

## Methods

### Data Availablity

The FINNPEC (Finnish Genetics of Pre-Eclampsia Consortium) data that support the findings of this study are available from Hannele Laivuori (hannele.laivuori@tuni.fi) on reasonable request. Additional review by the National Health Register Authorities and ethics committees may be required. Because of the sensitive nature of the FinnGen data collected for this study, requests to access the data set from qualified researchers trained in human subject confidentiality protocols may be sent to the Finnish Social and Health Data Permit Authority, Findata, at https://findata.fi/en/permits/. Aggregate-level FinnGen data from Data Freeze 12 is publicly available at https://www.finngen.fi/en/access_results.

### Study Population and Design

The study population consists of Finnish nationwide health registry data from 2 cohorts: the FINNPEC Study and the FinnGen study (Figure).

**Figure.**
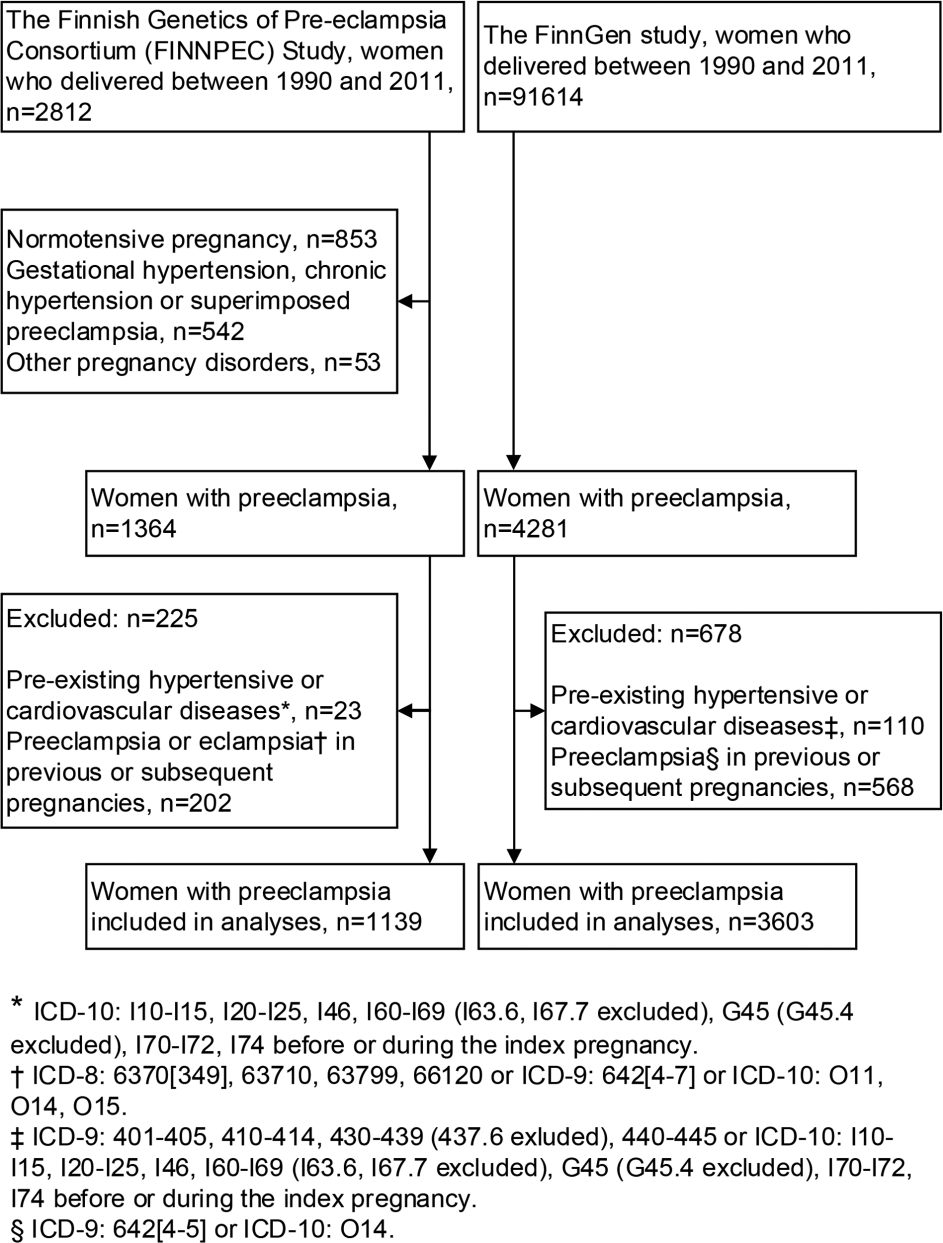
Flowchart of participant selection in the FINNPEC (Finnish Genetics of Pre-Eclampsia Consortium) case-control cohort and FinnGen cohort.

### FINNPEC

The FINNPEC case-control cohort includes 2812 nulliparous or multiparous women with a singleton pregnancy. In the prospectively recruited arm, women with preeclampsia (n=923) and women with non-preeclamptic pregnancies (n=1009) were identified at all 5 university hospitals in Finland from 2008 to 2011. After the recruitment of a woman with preeclampsia, a control woman was recruited from the same hospital. In the retrospectively recruited arm, all women diagnosed with preeclampsia or placental insufficiency during their pregnancies in 2000 to 2008 (except in Kuopio University Hospital, which recruited from 1990–2008) were retrospectively identified based on medical records and invited to participate. Women with a multiple pregnancy, age below 18 years, or an inability to provide informed consent in Finnish or Swedish were excluded. The FINNPEC Study has been described in further detail elsewhere.^21^ After the publication of the cohort profile, a small number of participants whose data were processed later were included. In total, our sample of the FINNPEC cohort includes 1364 participants with preeclampsia without prior hypertension.

The clinical data of the FINNPEC participants include information on maternal medical history, HDPs, and other obstetric complications in the index pregnancy, and maternal baseline characteristics. Information on FINNPEC participants’ CVD diagnoses during follow-up and HDP diagnoses in subsequent pregnancies was obtained from the Finnish Care Register for Health Care. The national population-based register contains digital health records from inpatient and specialized outpatient hospital data.

### FinnGen

The FinnGen study combines genotype data from Finnish nationwide biobanks with phenotype data from nationwide digital health registers. Reproductive history data, including information on pregnancy and delivery, were obtained from the Medical Birth Register and the Population Register. Information on CVD diagnoses was obtained from the Finnish Care Register for Health Care, including digital health records from inpatient and specialized outpatient care, and the Causes of Death register. All diagnosis dates have been randomized within a range of ±15 days. For each individual, the same number of days has been either added or subtracted from all their diagnosis dates. In addition to the register data, information on smoking and body mass index (BMI) was provided by biobanks. The study phenotypes, including preeclampsia, were based on the *International Classification of Diseases, Ninth and Tenth Revisions* (ICD-9 and ICD-10). This study sample was derived from FinnGen Data Freeze 12 (Aug 2023). A detailed description of the FinnGen study is provided elsewhere.^22^

### Definitions

In FINNPEC, preeclampsia was defined according to the American College of Obstetricians and Gynecologists 2002 criteria,^23^ as hypertension (systolic blood pressure ≥140 mm Hg and diastolic blood pressure ≥90 mm Hg) and proteinuria (urinary excretion of ≥0.3 g protein in a 24-hour specimen, or 0.3 g/L, or 2 ≥1+ readings on a dipstick in a random urine sample with no evidence of a urinary tract infection) occurring after 20^0/7^ wg. Each diagnosis was ascertained based on hospital records and confirmed independently by a research nurse and a study physician. In FinnGen, preeclampsia diagnosis was classified as 642.4 or 642.5 in the ICD-9 classification, and as O14 in the ICD-10 classification.

Two separate definitions for early-onset preeclampsia were used: preeclampsia was defined as early-onset when delivery or diagnosis occurred before 34^0/7^ wg and late-onset when at 34^0/7^ wg or later. In FINNPEC, preeclampsia was considered severe if one or more following criteria were present: systolic blood pressure ≥160 mm Hg and diastolic blood pressure ≥110 mm Hg, proteinuria ≥5 g/24 h, or subjective symptoms, and in FinnGen using ICD-9 codes 642.5 or 642.6 and ICD-10 codes O141 or O15. All other preeclampsia cases were defined as moderate. Delivery was defined as preterm when occurring before 37^0/7^ wg. A small for gestational age (SGA) infant was classified as birth weight at least 2 SD units below the mean for gestational age in FINNPEC and as an ICD-9 diagnosis 656.5 or an ICD-10 diagnosis O36.5 (maternal care for known or suspected poor fetal growth) in FinnGen. Placental insufficiency was defined as a pulsatility index or resistance index >+2 SD in umbilical arteries in fetal ultrasound.

### Exclusion Criteria

Only women with preeclampsia who delivered between 1990 and 2011 were included. In FINNPEC, each woman was classified into a single HDP category, meaning that chronic and gestational hypertension were automatically excluded. Exclusion criteria were preexisting CVDs before or during the index pregnancy. Additionally, women with preeclampsia in any other pregnancy were excluded to avoid confounding preeclampsia duration. In FinnGen, <18-year-old women and multiple pregnancies were also excluded, consistent with FINNPEC recruitment.

### Follow-Up Period

In statistical analyses, each participant’s follow-up period was defined as their age at the earliest of the following: a CVD diagnosis as the event, or censoring at the age of 55 years or at the closing date of monitoring (December 31, 2020) if CVD had not occurred. CVDs diagnosed at or over the age of 55 were excluded, as the prevalence of CVDs increases after menopause^24^ and most women have reached menopause by that age.^25^ Although the global prevalence of CVD has declined among individuals aged ≥55 years, the burden of disease has increased among younger adults aged 20 to 54 years.^26^

Follow-up data were available for all participants through national health registries. However, in FINNPEC, information on death or emigration during follow-up was not available; thus, individuals without diagnoses were assumed to be healthy. The number of such cases is presumably low in this under-55-year-old population. In FinnGen, no loss to follow-up occurred.

### Exposure and Outcomes

The exposure was the duration from preeclampsia diagnosis to delivery, measured in days. Preeclampsia duration was defined as 1 day if the preeclampsia diagnosis and delivery occurred on the same day. The outcome was a composite CVD outcome, including hypertensive diseases, ischemic heart diseases, cerebral/precerebral arterial diseases, and peripheral artery diseases diagnosed before the age of 55. The included diagnoses are presented in Table S1. In FINNPEC data, CVD diagnosis times were available with 1-year accuracy. In FinnGen, precise times, except for randomization of ±15 days, were available.

### Statistical Analyses

Maternal baseline characteristics were presented as mean and SD for normally distributed variables, median and interquartile ranges (IQR) for skewed variables, and absolute numbers and percentages for categorical variables. The normality of variables was assessed both graphically and with the Kolmogorov-Smirnov and Shapiro-Wilk tests.

The association between preeclampsia duration (in days) and composite CVD outcomes was assessed with the Cox proportional hazards model, using both univariable and multivariable models. Hazard ratios (HR) with 95% CIs were reported. The proportional hazards assumption was evaluated using Schoenfeld residuals.

Early-onset preeclampsia, preterm delivery, maternal age at delivery, pregestational BMI, primiparity, gestational diabetes, pregestational type 1 and 2 diabetes, SGA, placental insufficiency, preeclampsia in mother’s first-degree relatives, smoking, and sex of the child were considered possible confounders. BMI was included as a continuous variable and defined as weight in kilograms divided by the square of height in meters. Stepwise (backward and forward) selection was used for multivariable models. Subgroup analyses were conducted to assess the association between preeclampsia duration and CVD within the subgroups of severe and moderate preeclampsia.

FINNPEC and replication in FinnGen were analyzed separately due to data protection and differences in data collection and structure. Statistical analyses were performed with SPSS version 29 (IBM Corp., Armonk, NY) for FINNPEC data, and R version 4.4.2 for FinnGen data. Cases with missing data were excluded from the analyses. Statistical significance was defined as 2-tailed *P*<0.05.

## Results

### FINNPEC

There were 1364 women with preeclampsia identified from 1990 through 2011. Of these, 225 women were excluded for preexisting CVDs or preeclampsia in any previous or subsequent pregnancies. There were 1139 eligible participants with preeclampsia included in the analysis (Figure).

The median time from preeclampsia diagnosis to delivery was 9 days (IQR, 4–15 days, range 1–76 days). Delivery occurred before 34^0/7^ wg in 15.7% of the women, and 33.4% had preterm delivery. The mean maternal age at delivery was 30.1 years (SD, 5.3), and the median BMI before pregnancy was 23.5 kg/m^2^ (IQR, 21.2–26.8). Most women were primiparous (80.0%). Gestational diabetes was present in 11.9% of the women, and 2.6% had pregestational type 1 or 2 diabetes. SGA was common, occurring in 21.6% of the cases. (Table 1) The median age at the end of follow-up was 43.4 years (IQR, 39.3–48.6; range, 28.6–55.0 years), and the median follow-up time after delivery was 12.2 years (IQR, 11.0–15.5; range, 0.0–30.3 years). Composite CVD outcome occurred in 57 women (5.0% of the study population), and 36 of these had a hypertensive disease as their first diagnosis.

**Table 1.**
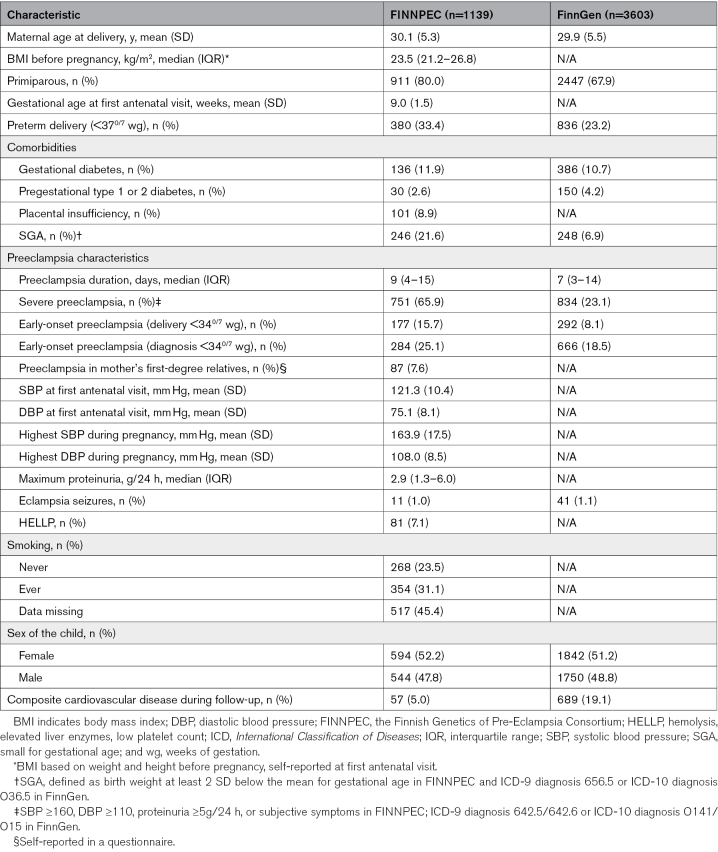
Baseline Characteristics of Women With a Diagnosis of Preeclampsia

Duration from preeclampsia diagnosis to delivery was positively associated with a composite maternal CVD: the crude HR for each day between diagnosis and delivery was 1.02 (95% CI, 1.00–1.04); *P*=0.020. In addition, the risk of CVD was higher among those with high BMI before pregnancy and low maternal age at delivery, and lower among those with SGA. The association between preeclampsia duration and CVD remained significant in the multivariable analysis adjusted for BMI, age at delivery, SGA, and smoking (adjusted HR, 1.02 [95% CI, 1.00–1.05]; *P*=0.022; identical model with backward and forward selection; Table 2). The association was stronger in an alternative model excluding smoking (Table S2).

**Table 2.**
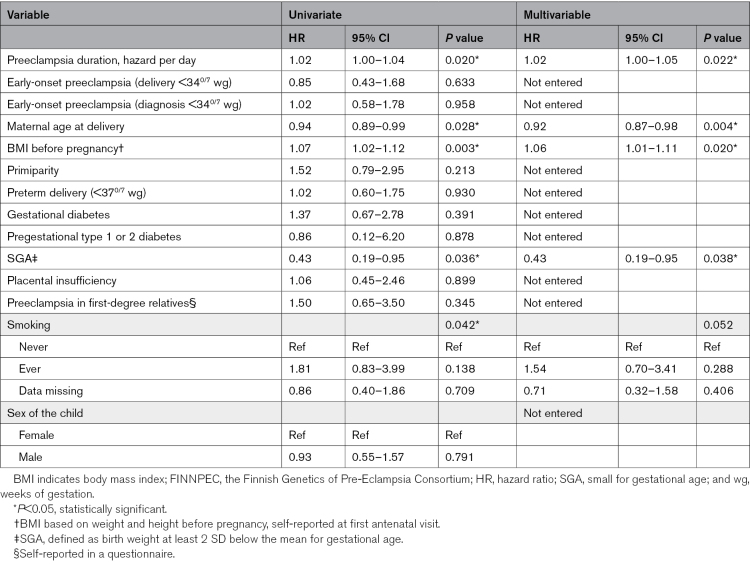
Risk Factors for Maternal Cardiovascular Diseases Before the Age of 55 in Women With a History of Preeclampsia in the FINNPEC Study (n=1139)

### FinnGen

There were 4281 women with preeclampsia identified from 1990 through 2011. Of these, 678 women were excluded for preexisting CVDs or preeclampsia in any previous or subsequent pregnancies. There were 3603 eligible participants with preeclampsia included in the analysis (Figure).

The median time from preeclampsia diagnosis to delivery was 7 days (IQR, 3–14 days; range, 1–158 days). Delivery occurred before 34^0/7^ wg in 8.1% of the women, and 23.2% had preterm delivery. The mean maternal age at delivery was 29.9 years (SD, 5.5), and 67.9% of the women were primiparous. Gestational diabetes was present in 10.7% and pregestational type 1 or 2 diabetes in 4.2% of the participants. SGA was observed in 6.9% of the cases. (Table 1) The median age at the end of follow-up was 48.4 years (IQR, 42.2–54.0; range, 20.4–55.0 years), and the median follow-up time after delivery was 17.1 years (IQR, 12.5–22.2; range, 0.0–31.1 years). Composite CVD outcome occurred in 689 women (19.1% of the study population). Of these, 671 had a hypertensive disease, and 18 had a peripheral artery disease. Ischemic heart disease or cerebral/precerebral arterial disease were not observed as first CVD diagnoses during the follow-up.

Duration from preeclampsia diagnosis to delivery was associated with a modestly increased risk of maternal CVDs: the crude HR for each day of latency was 1.01 (95% CI, 1.00–1.02); *P*<0.001. Women with early-onset preeclampsia, low maternal age at delivery, gestational diabetes, or pregestational type 1 or 2 diabetes had an elevated risk of CVD. The association between preeclampsia duration and CVD remained significant in multivariable analysis, adjusted for early-onset preeclampsia (delivery <34^0/7^ wg), maternal age at delivery, gestational diabetes, pregestational type 1 and 2 diabetes, and primiparity (adjusted HR, 1.01 [95% CI, 1.00–1.01]; *P*=0.012; backward selection; Table 3). Alternative multivariable models are presented in Table S3.

**Table 3.**
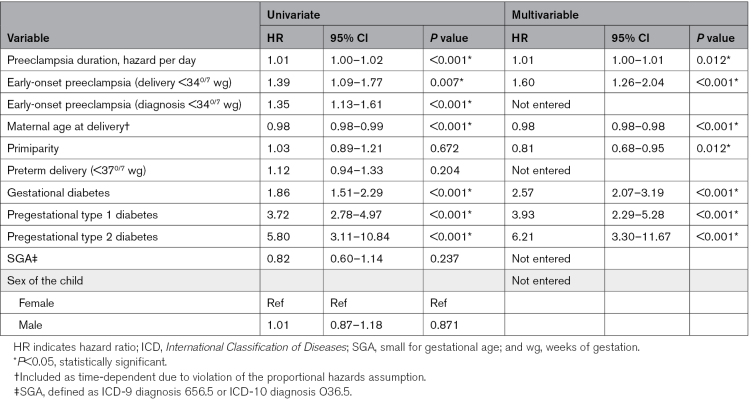
Risk Factors for Maternal Cardiovascular Diseases Before the Age of 55 in Women With History of Preeclampsia in the FinnGen Study (n=3603)

Pregestational BMI could not be included in the analyses due to the temporal inconsistency of maternal BMI measurements in the FinnGen cohort.

### Subgroup Analyses for Severe and Moderate Preeclampsia

In FINNPEC, longer preeclampsia duration was associated with a higher CVD risk in the severe preeclampsia subgroup (n=753; HR, 1.03 [95% CI, 1.01–1.05]; *P*=0.015), and the association remained significant in the multivariable model (Table S4). No association was observed in moderate preeclampsia (n=386; Table S5). Compared with moderate preeclampsia, women with severe preeclampsia had longer preeclampsia duration, were older at delivery, and more frequently experienced early-onset preeclampsia, preterm delivery, placental insufficiency, and SGA (Table S6).

In FinnGen, the duration of severe preeclampsia (n=834) was not associated with CVD risk (HR, 1.00 [95% CI, 0.99–1.01]; *P*=0.908; Table S7), whereas the positive association persisted in moderate preeclampsia (n=2769; HR, 1.01 [95% CI, 1.01–1.02]; *P*<0.001; Table S8). Severe preeclampsia women were more often primiparous and more frequently had early-onset preeclampsia, preterm delivery, SGA, and outcome CVD event. Preeclampsia duration did not differ significantly within the groups (Table S9).

## Discussion

Each additional day between preeclampsia diagnosis and delivery was associated with a 1% to 2% elevated risk of CVD before the age of 55, suggesting a dose-response relationship. In FINNPEC, this association persisted after adjusting for maternal age at delivery, BMI, SGA, and smoking, with each additional day corresponding to a 2% increase in CVD risk. The association was also significant in FinnGen when adjusted for early-onset preeclampsia (delivery <34^0/7^ wg), gestational diabetes, pregestational type 1 and 2 diabetes, primiparity, and maternal age.

Preeclampsia duration was more reliably defined in FINNPEC, where diagnosis dates were manually determined based on blood pressure and proteinuria measurements, whereas FinnGen data were based solely on health registries, potentially causing longer delays in diagnosis timing. This may explain the longer median preeclampsia duration in FINNPEC (9 days) compared with FinnGen (7 days). Consequently, FINNPEC results are likely more reliable.

The association between preeclampsia duration and long-term risk of maternal CVDs has rarely been studied. Two large cohort studies have shown a positive association between increasing preeclampsia duration and subsequent CVDs with median follow-up periods of 2.6 and 5.2 years after delivery, but contrary to our study, only preterm deliveries were included.^19,20^ Other studies have suggested that pregnancy prolongation in either early-onset, late preterm, or term preeclampsia pregnancies is not associated with increased risk of later CVDs in shorter follow-up periods.^16–18^ The significantly longer follow-up after delivery (12.2 years in FINNPEC and 17.1 years in FinnGen) in our study may explain our results.

Management strategies of HDPs have been assessed at different gestational ages.^15,27,28^ Planned early delivery after 34^0/7^ wg is associated with a decreased risk of adverse short-term maternal outcomes, such as HELLP syndrome (hemolysis, elevated liver enzymes, low platelet count) and eclampsia,^15,27^ and severe renal impairment, pulmonary edema, and abruptio placentae, compared with expectant management.^15^ On the contrary, planned early delivery after 34^0/7^ wg is associated with a significant risk of respiratory distress syndrome^15,27^ and neonatal intensive care unit admission.^15,27,28^ To improve perinatal outcomes, the Finnish Current Care Guidelines on hypertensive disorders of pregnancy and preeclampsia recommend delaying delivery until 37^0/7^ wg, or until 34^0/7^ wg if preeclampsia is severe.^29^ Although our findings suggest a potential benefit of early delivery after preeclampsia diagnosis in terms of long-term maternal cardiovascular outcomes, it is crucial to carefully consider the perinatal risks associated with early deliveries.

Unlike in previous studies investigating the association between preeclampsia duration and long-term CVD risk, all onset times of preeclampsia were included in our study. The association between preeclampsia and CVD is more pronounced in women with early-onset preeclampsia than among those with late-onset preeclampsia.^11,13^ Due to the sample size, early- and late-onset preeclampsia could not be stratified in our analyses. However, the FinnGen cohort showed that women with early-onset preeclampsia were at particularly high risk of CVDs, aligning with previous studies. The association between preeclampsia duration and CVD remained significant after adjusting for delivery before 34^0/7^ wg, suggesting that the effect is not fully mediated through early-onset preeclampsia. Nonetheless, when early-onset preeclampsia was defined by the time of diagnosis, the association attenuated (Table S3), indicating possible confounding.

In addition to preeclampsia, gestational hypertension is associated with increased CVD risk.^30^ However, the underlying pathophysiological mechanisms likely differ between the 2 conditions.^31^ Endothelial dysfunction, characterized by elevation of antiangiogenic markers and decrease of proangiogenic markers, is prominent in preeclampsia, whereas in gestational hypertension the changes appear only after 34 wg and remain milder throughout pregnancy.^32^ Additionally, there are differences in risk factors and pregnancy outcomes between the 2 conditions.^33^ For these reasons, we did not include isolated gestational hypertension as an exposure in this study.

In our data, each 1-year increase in maternal age at delivery was associated with a 2% to 6% reduction in CVD risk. However, this likely reflects the influence of related protective factors rather than age itself. Previous studies have shown that advanced age at pregnancy may have an adverse long-term effect on a woman’s cardiovascular health due to impaired adaptation to pregnancy.^34^ Nevertheless, when adjusted for socioeconomic, fertility, and health characteristics, no differences in long-term cardiovascular health were observed between women having their first birth at <35 versus ≥35 years.^35^ Conversely, advanced age at first birth may be protective, as higher socioeconomic status is positively associated with both advanced age at first birth and a reduced CVD risk.^36–38^ Preeclampsia inherently indicates poor cardiovascular adaptation during pregnancy,^34^ implying that all participants in our study already had impaired adaptation. This may enhance the relevance of socioeconomic factors in determining CVD risk within this group, though their exact contribution remains uncertain.

Other significant covariates included pregestational BMI (FINNPEC) and pregestational type 1 and 2 diabetes, as well as gestational diabetes (FinnGen). A higher BMI and type 1 and 2 diabetes were positively associated with CVD risk, aligning with prior research.^39–42^ Additionally, women with gestational diabetes were at higher risk of CVDs compared with those without gestational diabetes. Although gestational diabetes substantially predicts type 2 diabetes,^43^ it is also independently associated with an elevated CVD risk.^44,45^ These groups, as well as women with early-onset or long-lasting preeclampsia, or young age at delivery, should receive the most intense CVD prevention.

This study has a number of strengths. The clinical data were detailed, particularly in the FINNPEC cohort. Utilizing register data enabled a significantly longer follow-up compared with previous studies, with no loss to follow-up. Unlike in conventional longitudinal studies, follow-up data were available for all participants through national health registers. Our study population had relatively good geographic coverage in Finland, as FinnGen participants represent all regions of Finland, and FINNPEC participants were recruited at all 5 university hospitals in Finland. This is essential as CVD incidence rates differ between geographic regions in Finland.^46^

The main limitation of this study is the relatively small number of new-onset CVDs in the FINNPEC cohort. CVD rates differed between the cohorts: 57 (5.0%) in FINNPEC and 689 (19.1%) in FinnGen, likely due to differences in follow-up duration and maternal age at the end of follow-up. Both cohorts may underestimate CVD rates because the Finnish Care Register for Health Care contains medical records only from inpatient and specialized outpatient health care, excluding diagnoses from primary, occupational, or private health care services. Additionally, hypertension often remains undiagnosed, particularly in young and middle-aged adults.^47^ The FINNPEC cohort includes only patients from university hospitals and thus may represent more severe cases of preeclampsia compared with FinnGen. Furthermore, delays in diagnosing preeclampsia may occur in both cohorts, potentially leading to underestimation of preeclampsia duration.

In conclusion, the risk of hypertension and other CVDs occurring before the age of 55 increases by 1% to 2% for each day from preeclampsia diagnosis to delivery. The risk may be partially mediated through early-onset preeclampsia. Furthermore, those who deliver at a young age, have high BMI, or have pregestational or gestational diabetes are at particularly high risk.

## Perspectives

Considering their long-term cardiovascular health, women with preeclampsia might potentially benefit from early delivery. Future research investigating the mechanisms of endothelial dysfunction in women exposed to preeclampsia is needed to identify pathophysiological links to CVDs. However, women with a history of preeclampsia are not a homogeneous group regarding their long-term cardiovascular risk. While individuals with obesity or diabetes typically receive CVD counseling, our findings suggest that targeted prevention should also be extended to young women with a history of preeclampsia, especially if the condition had an early gestational age of onset or persisted for a long period. Identifying these high-risk individuals and providing tailored CVD prevention may help mitigate their long-term cardiovascular risks.

### Appendix

Collaborative Authorship Groups

FINNPEC Core Investigator Group

Hannele Laivuori, Seppo Heinonen, Eero Kajantie, Juha Kere, Katja Kivinen, Anneli Pouta

FinnGen

Aarno Palotie, Mark Daly, Bridget Riley-Gills, Howard Jacob, Coralie Viollet, Slavé Petrovski, Alix Berton, Santha Ramakrishnan, Ellen Tsai, Zhihao Ding, Emily Holzinger, Robert Plenge, Joseph Maranville, Mark McCarthy, Rion Pendergrass, Jonathan Davitte, Chia-Yen Chen, Melis Atalar Aksit, Anna Vlahiotis, Katherine Klinger, Clement Chatelain, Jorg Blankenstein, Karol Estrada, Robert Graham, Dawn Waterworth, Chris O´Donnell, Nicole Renaud, Tomi P. Mäkelä, Jaakko Kaprio, Minna Ruddock, Lila Kallio, Antti Hakanen, Terhi Kilpi, Markus Perola, Jukka Partanen, Taneli Raivio, Eero Punkka, Teija Kekonen, Raisa Serpi, Kati Kristiansson, Sanna Siltanen, Veli-Matti Kosma, Arto Mannermaa, Jari Laukkanen, Tiina Jokela, Mervi Ahlroth, Johanna Mäkelä, Outi Tuovila, Jeffrey Waring, Fedik Rahimov, Ioanna Tachmazidou, Marc Jung, Hanati Tuoken, Shameek Biswas, Benjamin Sun, Neha Raghavan, Jae-Hoon Sul, Xinli Hu, Ma´en Obeidat, Jonathan Chung, Jonas Zierer, Mari Niemi, Samuli Ripatti, Johanna Schleutker, Tiina Wahlfors, Mikko Arvas, Olli Carpén, Reetta Hinttala, Johannes Kettunen, Katriina Aalto-Setälä, Mika Kähönen, Hanna Kujala, Triin Laisk, Natalia Pujol, Veikko Salomaa, Jaana Suvisaari, Satu Koskela, Jouni Lauronen, Kristiina Aittomäki, Pirkko Pussinen, Tuomo Meretoja, Heikki Joensuu, Peeter Karihtala, Emma Juuri, Aino Salminen, Tuula Salo, David Rice, Pekka Nieminen, Ulla Palotie, Fredrik Åberg, Daniel Gordin, Patrik Finne, Joni A Turunen, Minna Raivio, Pentti Tienari, Martti Färkkilä, Jukka Koskela, Sampsa Pikkarainen, Kari Eklund, Paula Kauppi, Juha Sinisalo, Marja-Riitta Taskinen, Tiinamaija Tuomi, Timo Hiltunen, Johanna Mattson, Eveliina Salminen, Terhi Ollila, Katariina Hannula-Jouppi, Oskari Heikinheimo, Ilkka Kalliala, Lauri Aaltonen, Erkki Isometsä, Antti Aarnisalo, Ilkka Immonen, Salla Ranta, Filip Scheperjans, Felix Vaura, Nina Mars, Esa Pitkänen, Hannele Laivuori, Katja Kivinen, Elisabeth Widen, Taru Tukiainen, Hanna Ollila, Elmo Saarentaus, Anne Kerola, Eero Vuoksimaa, Joni Lindbohm, Zhiyu Yang, Matthew Sampson, Adrian Banerji, Michelle McNulty, Aoxing Liu, Joel Rämö, Austin Argentieri, Amanda Elliott, Elisa Rahikkala, Kirsi Sipilä, Valtteri Julkunen, Ville Leinonen, Sanna Toppila-Salmi, Mikko Hiltunen, Eino Solje, Hannu Kankaanranta, Antti Mäkitie, Iiris Hovatta, Niko Välimäki, Minttu Marttila, Anne Portaankorva, Eija Laakkonen, Heidi Silven, Eeva Sliz, Riikka Arffman, Susanna Savukoski, Riitta Kaarteenaho, Jaakko Tyrmi, Laura Kuusalo, Laura Pirilä, Tapio Hellman, Matti Vuori, Teemu Niiranen, Timo Blomster, Johanna Huhtakangas, Terttu Harju, Kaisa Tasanen, Laura Huilaja, Vuokko Anttonen, Marja Vääräsmäki, Outi Uimari, Laure Morin-Papunen, Maarit Niinimäki, Terhi Piltonen, Reetta Kälviäinen, Hilkka Soininen, Mikko Kiviniemi, Oili Kaipiainen-Seppänen, Margit Pelkonen, Päivi Auvinen, Maria Siponen, Liisa Suominen, Päivi Mäntylä, Kai Kaarniranta, Jukka Peltola, Airi Jussila, Katri Kaukinen, Pia Isomäki, Jussi Hernesniemi, Annika Auranen, Hannu Uusitalo, Teea Salmi, Venla Kurra, Laura Kotaniemi-Talonen, Argyro Bizaki-Vallaskangas, Juha Rinne, Roosa Kallionpää, Markku Voutilainen, Antti Palomäki, Riitta Lahesmaa, Kaj Metsärinne, Jenni Aittokallio, Klaus Elenius, Sirkku Peltonen, Leena Koulu, Ulvi Gursoy, Varpu Jokimaa, Tytti Willberg, Adam Ziemann, Nizar Smaoui, Anne Lehtonen, Apinya Lertratanakul, Relja Popovic, Mengzhen Liu, Anneke Den Hollander, Jan Freudenberg, Britney Milkovich, Andrew Blumenfeld, Tushar Kumar, Dirk Paul, Bram Prins, Eleanor Wheeler, Kousik Kundu, Santosh Atanur, Andrew Lowe, Thomas Spargo, Oliver Burren, Margarete Fabre, Fabio Baschiera, Hans van Leeuwen, Himanshu Manchanda, Karl Heilbron, Martin Rao, Nicole Schmidt, Samu Kurki, Johanna Mielke, Juho Immonen, Thomas Battram, Tobias Hogrebe, Susan Eaton, Ketian Yu, Stephanie Loomis, Coro Paisan-Ruiz, Elke Markert, Frank Li, Yao Hu, Christoph Ogris, Eric Simon, Julio Cesar Bolivar Lopez, Monika Frysz, Marla Hochfeld, Cara Carty, Michael Turchin, Neelakshi Jog, Corneliu Bodea, Janie Shelton, Chen Li, Kritika Singh, Peng Jiang, Elena Sanchez, Lilith Moss, Zijie Zhao, Anna Podgornaia, Natalie Bowers, Edmond Teng, Tim Lu, Hubert Chen, Jennifer Schutzman, Erich Strauss, Hao Chen, David Choy, Brian Yaspan, Cameron Adams, Michael Rothenberg, Sergio Dellepiane, Michael Holmes, Diana Chang, Tushar Bhangale, Fanli Xu, Laura Addis, John Eicher, Linda McCarthy, Jorge Esparza Gordillo, Joanna Betts, Rajashree Mishra, Audrey Chu, Diptee Kulkarni, Janet Kumar, Charli Harlow, Lea Sarow-Blat, Diana L.Cousminer, Jagtar Nijjar, Jessica Chao, Michal Magid, Shashank Jariwala, Chris Floyd, Dan Swerdlow, Erding Hu, Prerak Desai, Stephen Haddad, Damien Croteau-Chonka, Billy Fahy, Paola Bronson, Kirsi Auro, David Pulford, Sauli Vuoti, Dermot Reilly, Karen He, Ekaterina Khramtsova, Amy Hart, Meijian Guan, Alessandro Porello, P. Dunnmon, Sara Gale, Brice Keyes, John Kwon, Jonathan Sherlock, Matt Loza, Chris Whelan, W Galpern, Yanfei Zhang, Mona Selej, Abolfazl Doostparast Torshizi, Qingqin S Li, Sahar Mozzafari, Christopher Deboever, Jason Miller, Fabiana Farias, Andrey Loboda, Jorge Del-aguila, Elisabeth Vollmann, Jozsef Karman, Julie Fiore, Rajesh Kamath, Andrei Popescu, Delphine Fagegaltier, Travis Barr, Aristide Merola, Oliver Freeman, Simonne Longerich, Enrico Ferrero, Nikos Patsopoulos, Nancy Finkel, Sabina Pfister, Shola Richards, Katherine Mccauley, Xiaobo Xia, Mike Mendelson, Majd Mouded, Debby Ngo, Kirsi Kalpala, Melissa Miller, Nan Bing, Jaakko Parkkinen, Heli Lehtonen, Stefan McDonough, Ying Wu, Erin Macdonald-Dunlop, Jessica Chung, Michael McLean, Joshua Chiou, Hye In Kim, Sivakumar Pitchumani, Sumedha Jassal, Madhurima Saxena, Catherine O’Riordan, Samuel Lessard, Suzanne Jacobs, Hamid Mattoo, David Habiel, Guanling Huan, Anu Jalanko, Risto Kajanne, Mervi Aavikko, Helen Cooper, Denise Öller, Tarja Laitinen, Sofia Kuitunen, Auli Toivola, Rodos Rodosthenous, Mitja Kurki, Juha Karjalainen, Pietro Della Briotta Parolo, Arto Lehisto, Juha Mehtonen, Reza Jabal, Mutaamba Maasha, Sanni Ruotsalainen, Samuel Jones, Raymond Walters, Paavo Häppölä, L. Elisa Lahtela, Johanna Paltta, Juulia Partanen, Mari Kaunisto, Elina Kilpeläinen, Tianduanyi Wang, Timo P. Sipilä, Oluwaseun Alexander Dada, Awaisa Ghazal, Rigbe Weldatsadik, Jaska Uimonen, Kati Donner, Päivi Laiho, Susanna Lemmelä, Teemu Paajanen, Arto Pietilä, Aki Havulinna, Mary Pat Reeve, Shanmukha Sampath Padmanabhuni, Harri Siirtola, Javier Gracia-Tabuenca, Marika Kaakinen, Shuang Luo, Vincent Llorens, Dawit Yohannes, Iina Laak, Pauli Wihuri, Tom Southerington, Meri Lähteenmäki

## ARTICLE INFORMATION

### Acknowledgments

The authors thank Eija Kortelainen for expert technical assistance, and the members, assisting personnel, and participants of the FINNPEC (Finnish Genetics of Pre-Eclampsia Consortium) Study, as well as the participants and investigators of the FinnGen study (see Supplemental Material), for their valuable contributions to this work. Following biobanks are acknowledged for delivering biobank samples to FinnGen: Auria Biobank (www.auria.fi/biopankki), Finnish Institute for Health and Welfare Biobank (www.thl.fi/biobank), Helsinki Biobank (www.helsinginbiopankki.fi), Biobank Borealis of Northern Finland (https://oys.fi/en/front-page/for-researchers/biobank-borealis-of-northern-finland/), Finnish Clinical Biobank Tampere (https://www.pirha.fi/en/web/english/for-professionals/finnish-clinical-biobank-tampere), Biobank of Eastern Finland (www.ita-suomenbiopankki.fi/en), Central Finland Biobank (www.ksshp.fi/fi-FI/Potilaalle/Biopankki), Finnish Red Cross Blood Service Biobank (www.veripalvelu.fi/verenluovutus/biopankkitoiminta), and Arctic Biobank (https://www.oulu.fi/en/university/faculties-and-units/faculty-medicine/northern-finland-birth-cohorts-and-arctic-biobank). All Finnish Biobanks are members of BBMRI.fi infrastructure (https://www.bbmri-eric.eu/national-nodes/finland/). Finnish Biobank Cooperative (https://finbb.fi/) is the coordinator of Biobanking and Biomolecular Resources Research Infrastructure – European Research Infrastructure Consortium operations in Finland. The Finnish biobank data can be accessed through the Fingenious services (https://site.fingenious.fi/en/) managed by Finnish Biobank Cooperative.

During the preparation of this article, N. Keitaanpää used ChatGPT-4 to enhance the style and readability of self-written text. After using artificial intelligence, N. Keitaanpää reviewed and revised the content as needed and takes full responsibility for the content of the publication.

### Sources of Funding

This study was supported by Jane and Aatos Erkko Foundation (to H. Laivuori); the Päivikki and Sakari Sohlberg Foundation (to A. Kivelä and H. Laivuori); Academy of Finland (362074, 121196, 278941, 134957 to H. Laivuori); Research Funds of the University of Helsinki (to H. Laivuori); The Finnish Medical Foundation (to H. Laivuori); Finska Läkaresällskapet (to H. Laivuori); the Juho Vainio Foundation (to T. Jääskeläinen), EraPerMed JTC2020, Academy of Finland (344695, to H. Laivuori); the Competitive State Research Financing of the Expert Responsibility area of Helsinki University Hospital (to S. Heinonen), and Tampere University Hospital (to H. Laivuori).

The Novo Nordisk Foundation, the Signe and Ane Gyllenberg Foundation, and the Foundation for Pediatric Research contributed to the FINNPEC (Finnish Genetics of Pre-Eclampsia Consortium) Study. The FinnGen project is funded by 2 grants from Business Finland (HUS 4685/31/2016 and UH 4386/31/2016) and the following industry partners: AbbVie Inc., AstraZeneca UK Ltd, Biogen MA Inc., Bristol Myers Squibb Inc. (And Celgene Corporation & Celgene International II Sàrl), Genentech Inc., Merck Sharp & Dohme LCC, Pfizer Inc., GlaxoSmithKline Intellectual Property Development Ltd., Sanofi US Services Inc., Maze Therapeutics Inc., Johnson&Johnson Innovative Medicine Inc., Novartis AG, Boehringer Ingelheim International GmbH and Bayer AG.

### Disclosures

H. Laivuori and T. Jääskeläinen have received honoraria from Orion Corporation. The other authors report no conflicts.

### Supplemental Material

Ethical Considerations

Supplementary Methods

Tables S1–S9

Collaborative Authorship Groups

## Supplementary Material


