# Supplementary material for: Impact of Preeclampsia Duration on Long-Term Cardiovascular Disease Risk
Source: Hypertension. 2025 Nov 18;83(2):e25054. doi: 10.1161/HYPERTENSIONAHA.125.25054 (PMC12822770; doi:10.1161/HYPERTENSIONAHA.125.25054)
Supplement: Supplementary file 1 [file hyp-83-e25054-s001.docx]

**Supplemental Online Material**

**Impact of Preeclampsia Duration on Long-Term Cardiovascular Disease Risk**

**Short title:** Preeclampsia Duration and Cardiovascular Disease

Nina Keitaanpää^1^, Jaakko S. Tyrmi, PhD^1,2^, Elli Toivonen, MD, PhD^1,3^, Heini Huhtala, MSc^4^, Anni Kivelä, MSc^5^, FINNPEC Core Investigator Group, FinnGen, Seppo Heinonen, MD, PhD^6^, Tiina Jääskeläinen, PhD^5,7^, Hannele Laivuori, MD, PhD^1,3,5,8^

^1^Center for Child, Adolescent, and Maternal Health Research, Faculty of Medicine and Health Technology, Tampere University, Tampere, Finland

^2^Center for Life Course Health Research, Faculty of Medicine, University of Oulu, Oulu, Finland

^3^Department of Obstetrics and Gynecology, Tampere University Hospital, The Wellbeing Services County of Pirkanmaa, Finland

^4^Faculty of Social Sciences, Tampere University, Tampere, Finland

^5^Medical and Clinical Genetics, University of Helsinki and Helsinki University Hospital, Helsinki, Finland

^6^Department of Obstetrics and Gynecology, Helsinki University Hospital and University of Helsinki, Helsinki, Finland

^7^Department of Food and Nutrition, University of Helsinki, Helsinki, Finland

^8^Institute for Molecular Medicine Finland, Helsinki Institute of Life Science, HiLIFE, University of Helsinki, Helsinki, Finland

**Correspondence:**

Nina Keitaanpää, [nina.keitaanpaa@tuni.fi](mailto:nina.keitaanpaa@tuni.fi)

Arvo Ylpön katu 34

P.O. Box 100

FI-33014 Tampere University, Finland

**Ethical Considerations**

**Supplementary Methods**

**Table S1.** Definitions of cardiovascular diseases in the study according to the International Classification of Diseases, Ninth and Tenth Revisions (ICD-9 and ICD-10).

**Table S2.** Alternative multivariable model for maternal cardiovascular disease risk in women with history of preeclampsia in the FINNPEC Study (n=1139).

**Table S3.** Alternative multivariable models for maternal cardiovascular disease risk in women with history of preeclampsia in the FinnGen study (n=3603).

**Table S4.** Subgroup analysis: risk factors for maternal cardiovascular diseases in women with history of severe preeclampsia in the FINNPEC Study (n=386).

**Table S5.** Subgroup analysis: risk factors for maternal cardiovascular diseases in women with history of moderate preeclampsia in the FINNPEC Study (n=386).

**Table S6.** Baseline characteristics of women with a diagnosis of severe or moderate preeclampsia in the FINNPEC Study.

**Table S7.** Subgroup analysis: risk factors for maternal cardiovascular diseases in women with history of severe preeclampsia in the FinnGen study (n=2769).

**Table S8.** Subgroup analysis: risk factors for maternal cardiovascular diseases in women with history of moderate preeclampsia in the FinnGen study (n=2769).

**Table S9.** Baseline characteristics of women with a diagnosis of severe or moderate preeclampsia in the FinnGen study.

**Collaborative Authorship Groups**

FINNPEC Core Investigator Group

FinnGen

**Ethical Considerations**

The FINNPEC Study was approved by the Coordinating Ethics Committee of the Hospital District of Helsinki and Uusimaa (permit number 149/EO/2007). All FINNPEC Study participants and the participating parents of the neonates provided a written informed consent.

Study subjects in FinnGen provided informed consent for biobank research, based on the Finnish Biobank Act. Alternatively, separate research cohorts, collected prior the Finnish Biobank Act came into effect (in September 2013) and start of FinnGen (August 2017), were collected based on study-specific consents and later transferred to the Finnish biobanks after approval by Fimea (Finnish Medicines Agency), the National Supervisory Authority for Welfare and Health. Recruitment protocols followed the biobank protocols approved by Fimea. The Coordinating Ethics Committee of the Hospital District of Helsinki and Uusimaa (HUS) statement number for the FinnGen study is Nr HUS/990/2017.

The FinnGen study is approved by Finnish Institute for Health and Welfare (permit numbers: THL/2031/6.02.00/2017, THL/1101/5.05.00/2017, THL/341/6.02.00/2018, THL/2222/6.02.00/2018, THL/283/6.02.00/2019, THL/1721/5.05.00/2019 and THL/1524/5.05.00/2020), Digital and population data service agency (permit numbers: VRK43431/2017-3, VRK/6909/2018-3, VRK/4415/2019-3), the Social Insurance Institution (permit numbers: KELA 58/522/2017, KELA 131/522/2018, KELA 70/522/2019, KELA 98/522/2019, KELA 134/522/2019, KELA 138/522/2019, KELA 2/522/2020, KELA 16/522/2020), Findata permit numbers THL/2364/14.02/2020, THL/4055/14.06.00/2020, THL/3433/14.06.00/2020, THL/4432/14.06/2020, THL/5189/14.06/2020, THL/5894/14.06.00/2020, THL/6619/14.06.00/2020, THL/209/14.06.00/2021, THL/688/14.06.00/2021, THL/1284/14.06.00/2021, THL/1965/14.06.00/2021, THL/5546/14.02.00/2020, THL/2658/14.06.00/2021, THL/4235/14.06.00/2021, Statistics Finland (permit numbers: TK-53-1041-17 and TK/143/07.03.00/2020 (earlier TK-53-90-20) TK/1735/07.03.00/2021, TK/3112/07.03.00/2021) and Finnish Registry for Kidney Diseases permission/extract from the meeting minutes on 4^th^ July 2019.

The Biobank Access Decisions for FinnGen samples and data utilized in FinnGen Data Freeze 12 include: THL Biobank BB2017_55, BB2017_111, BB2018_19, BB_2018_34, BB_2018_67, BB2018_71, BB2019_7, BB2019_8, BB2019_26, BB2020_1, BB2021_65, Finnish Red Cross Blood Service Biobank 7.12.2017, Helsinki Biobank HUS/359/2017, HUS/248/2020, HUS/430/2021 §28, §29, HUS/150/2022 §12, §13, §14, §15, §16, §17, §18, §23, §58, §59, HUS/128/2023 §18, Auria Biobank AB17-5154 and amendment #1 (August 17 2020) and amendments BB_2021-0140, BB_2021-0156 (August 26 2021, Feb 2 2022), BB_2021-0169, BB_2021-0179, BB_2021-0161, AB20-5926 and amendment #1 (April 23 2020) and it´s modifications (Sep 22 2021), BB_2022-0262, BB_2022-0256, Biobank Borealis of Northern Finland_2017_1013, 2021_5010, 2021_5010 Amendment, 2021_5018, 2021_5018 Amendment, 2021_5015, 2021_5015 Amendment, 2021_5015 Amendment_2, 2021_5023, 2021_5023 Amendment, 2021_5023 Amendment_2, 2021_5017, 2021_5017 Amendment, 2022_6001, 2022_6001 Amendment, 2022_6006 Amendment, 2022_6006 Amendment, 2022_6006 Amendment_2, BB22-0067, 2022_0262, 2022_0262 Amendment, Biobank of Eastern Finland 1186/2018 and amendment 22§/2020, 53§/2021, 13§/2022, 14§/2022, 15§/2022, 27§/2022, 28§/2022, 29§/2022, 33§/2022, 35§/2022, 36§/2022, 37§/2022, 39§/2022, 7§/2023, 32§/2023, 33§/2023, 34§/2023, 35§/2023, 36§/2023, 37§/2023, 38§/2023, 39§/2023, 40§/2023, 41§/2023, Finnish Clinical Biobank Tampere MH0004 and amendments (21.02.2020 & 06.10.2020), BB2021-0140 8§/2021, 9§/2021, §9/2022, §10/2022, §12/2022, 13§/2022, §20/2022, §21/2022, §22/2022, §23/2022, 28§/2022, 29§/2022, 30§/2022, 31§/2022, 32§/2022, 38§/2022, 40§/2022, 42§/2022, 1§/2023, Central Finland Biobank 1-2017, BB_2021-0161, BB_2021-0169, BB_2021-0179, BB_2021-0170, BB_2022-0256, BB_2022-0262, BB22-0067, Decision allowing to continue data processing until 31^st^ Aug 2024 for projects: BB_2021-0179, BB22-0067,BB_2022-0262, BB_2021-0170, BB_2021-0164, BB_2021-0161, and BB_2021-0169, and Terveystalo Biobank STB 2018001 and amendment 25^th^ Aug 2020, Finnish Hematological Registry and Clinical Biobank decision 18^th^ June 2021, Arctic biobank P0844: ARC_2021_1001.

**Supplementary Methods**

Differences in baseline characteristics between severe and moderate preeclampsia were compared using independent-samples *t*-tests for normally distributed continuous variables, Mann–Whitney *U*-tests for skewed continuous variables, chi-square tests for categorical variables with frequencies >20, and Fisher’s exact tests for categorical variables with frequencies ≤20. Normality of continuous variables was evaluated both graphically and using the Kolmogorov–Smirnov and Shapiro–Wilk tests.

| **Table S1.** Definitions of cardiovascular diseases in the study according to the International Classification of Diseases, Ninth and Tenth Revisions (ICD-9 and ICD-10). | | |
| --- | --- | --- |
| Disease category | ICD-9 | ICD-10 |
| Hypertensive diseases | 401–405 | I10–I15 |
| Ischemic heart diseases, including all types of angina pectoris and acute cardiac arrest | 410–414 | I20–I25, I46 |
| Cerebral/precerebral arterial diseases | 430–439 (437.6 excluded) | I60–I69 (I63.6, I67.7 excluded), G45 (G45.4 excluded) |
| Peripheral artery diseases | 440–445 | I70–I72, I74 |

| **Table S2.** Alternative multivariable model for maternal cardiovascular disease risk in women with history of preeclampsia in the FINNPEC Study (n=1139). | | | | |
| --- | --- | --- | --- | --- |
| Variable | HR | 95% CI | *P*-value |  |
| Preeclampsia duration, hazard per day | 1.03 | 1.01–1.05 | **0.016** |  |
| Maternal age at delivery | 0.93 | 0.88–0.98 | **0.008** |  |
| BMI* before pregnancy | 1.06 | 1.02–1.11 | **0.009** |  |
| SGA† | 0.93 | 0.88–0.98 | **0.008** |  |
| * BMI, Body mass index, based on weight and height before pregnancy, self-reported at first antenatal visit.  † SGA, Small for gestational age infant. Defined as birth weight at least 2 SD below the mean for gestational age.  FINNPEC, the Finnish Genetics of Pre-eclampsia Consortium; HR, hazard ratio; CI, confidence interval. | | | | |

| **Table S3.** Alternative multivariable models for maternal cardiovascular disease risk in women with history of preeclampsia in the FinnGen study (n=3603). | | | | | | | | | | | | | | | | |
| --- | --- | --- | --- | --- | --- | --- | --- | --- | --- | --- | --- | --- | --- | --- | --- | --- |
| Variable | | Model 1 | | |  | Model 2 | | | | |  | | Model 3 | | | |
|  | | HR | 95% CI | *P*-value | | |  | HR | 95% CI | *P*-value | |  | | HR | 95% CI | *P*-value |
| Preeclampsia duration, hazard per day | | 1.01 | 1.00–1.01 | **0.015** | | |  | 1.01 | 1.00–1.01 | 0.082 | |  | | 1.00 | 1.00–1.01 | 0.345 |
| Early-onset preeclampsia (delivery <34^0/7^ wg) | | 1.49 | 1.17–1.90 | **0.001** | | |  | 1.49 | 1.07–2.08 | **0.018** | |  | | Not entered |  |  |
| Early-onset preeclampsia (diagnosis <34^0/7^ wg) | | Not entered |  |  | | |  | 1.07 | 0.81–1.40 | 0.631 | |  | | 1.32 | 1.08–1.61 | **0.007** |
| Maternal age at delivery* | | 0.99 | 0.98–1.00 | **0.004** | | |  | 0.98 | 0.98–0.99 | **<0.001** | |  | | 0.98 | 0.98–0.99 | **<0.001** |
| Gestational diabetes | | 2.22 | 1.79–2.75 | **<0.001** | | |  | 2.60 | 2.10–3.23 | **<0.001** | |  | | 2.57 | 2.07–3.18 | **<0.001** |
| Pregestational type 1 diabetes | | 3.94 | 2.93–5.28 | **<0.001** | | |  | 3.84 | 2.86–5.16 | **<0.001** | |  | | 3.75 | 2.79–5.03 | **<0.001** |
| Pregestational type 2 diabetes | | 5.87 | 3.12–11.07 | **<0.001** | | |  | 6.51 | 3.46–12.23 | **<0.001** | |  | | 6.40 | 3.41–12.03 | **<0.001** |
|  | Model 1: forward selection; Models 2–3: forced-entry (enter) including (2) all covariates significant in univariate analyses, (3) early-onset preeclampsia defined by timing of diagnosis.  * Included as time-dependent due to violation of proportional hazards assumption.  HR, hazard ratio; CI, confidence interval; wg, weeks of gestation. | | | | | | | | | | | | | | | |

| **Table S4.** Subgroup analysis: risk factors for maternal cardiovascular diseases in women with history of severe preeclampsia in the FINNPEC Study (n=753). | | | | | | | |
| --- | --- | --- | --- | --- | --- | --- | --- |
| Variable | Univariate | | | Multivariable* | | | |
|  | HR | 95% CI | *P*-value |  | HR | 95% CI | *P*-value |
| Preeclampsia duration, hazard per day | 1.03 | 1.01–1.05 | **0.015** |  | 1.02 | 1.00–1.05 | **0.039** |
| Early-onset preeclampsia (delivery <34^0/7^ wg) | 0.85 | 0.42–1.73 | 0.653 |  | Not entered |  |  |
| Early-onset preeclampsia (diagnosis <34^0/7^ wg) | 1.08 | 0.59–1.98 | 0.811 |  | Not entered |  |  |
| Maternal age at delivery | 0.96 | 0.90–1.01 | 0.133 |  | 0.94 | 0.89–1.00 | 0.050 |
| BMI† before pregnancy | 1.07 | 1.02–1.12 | **0.009** |  | 1.06 | 1.01–1.12 | **0.015** |
| Primiparity | 1.43 | 0.66–3.10 | 0.361 |  | Not entered |  |  |
| Preterm delivery (<37^0/7^ wg) | 1.03 | 0.56–1.87 | 0.935 |  | Not entered |  |  |
| Gestational diabetes | 1.29 | 0.57–2.89 | 0.544 |  | Not entered |  |  |
| Pregestational type 1 or 2 diabetes | 0.05 | 0.00–369.27 | 0.506 |  | Not entered |  |  |
| SGA‡ | 0.46 | 0.20–1.03 | 0.059 |  | Not entered |  |  |
| Placental insufficiency | 1.21 | 0.51–2.88 | 0.660 |  | Not entered |  |  |
| Preeclampsia in first-degree relatives§ | 2.38 | 1.01–5.65 | **0.049** |  | 2.36 | 0.99–5.60 | 0.052 |
| Smoking** |  |  | **0.003** |  | Not entered |  |  |
| Never | Ref | Ref | Ref |  |  |  |  |
| Ever | 1.92 | 0.83–4.41 | 0.126 |  |  |  |  |
| Data missing | 0.59 | 0.25–1.40 | 0.233 |  |  |  |  |
| Sex of the child |  |  |  |  | Not entered |  |  |
| Female | Ref | Ref | Ref |  |  |  |  |
| Male | 1.02 | 0.56–1.86 | 0.959 |  |  |  |  |
| * Backward and forward selection resulted in identical models.  † BMI, Body mass index, based on weight and height before pregnancy, self-reported at first antenatal visit.  ‡ SGA, Small for gestational age infant. Defined as birth weight at least 2 SD below the mean for gestational age.  § Self-reported in a questionnaire.  ** Smoking was considered for inclusion in backward and forward selection but violated the proportional hazards assumption in the multivariable model and was therefore not included.  FINNPEC, the Finnish Genetics of Pre-eclampsia Consortium; HR, hazard ratio; CI, confidence interval; wg, weeks of gestation; Ref, reference. | | | | | | | |

| **Table S5.** Subgroup analysis: risk factors* for maternal cardiovascular diseases in women with history of moderate preeclampsia in the FINNPEC Study (n=386). | | | | |
| --- | --- | --- | --- | --- |
| Variable | HR | 95% CI | *P*-value |  |
| Preeclampsia duration, hazard per day | 1.01 | 0.96–1.05 | 0.836 |  |
| Early-onset preeclampsia (delivery <34^0/7^ wg) | 0.05 | 0.00–2328.53 | 0.576 |  |
| Early-onset preeclampsia (diagnosis <34^0/7^ wg) | 0.04 | 0.00–97.73 | 0.422 |  |
| Maternal age at delivery | 0.88 | 0.78–1.00 | **0.044** |  |
| BMI† before pregnancy | 1.07 | 0.97–1.17 | 0.168 |  |
| Primiparity | 1.61 | 0.45–5.80 | 0.468 |  |
| Preterm delivery (<37^0/7^ wg) | 0.49 | 0.06–3.76 | 0.493 |  |
| Gestational diabetes | 1.63 | 0.36–7.33 | 0.524 |  |
| Pregestational type 1 or 2 diabetes | 5.45 | 0.67–44.28 | 0.113 |  |
| SGA‡ | 0.04 | 0.00-55.17 | 0.385 |  |
| Placental insufficiency | 0.05 | 0.00-939.09 | 0.542 |  |
| Preeclampsia in first-degree relatives§ | 0.04 | 0.00-107.86 | 0.430 |  |
| Smoking |  |  | 0.395 |  |
| Never | Ref | Ref | Ref |  |
| Ever | 1.45 | 0.13-16.08 | 0.760 |  |
| Data missing | 3.14 | 0.39-25.14 | 0.280 |  |
| Sex of the child |  |  |  |  |
| Female | Ref | Ref | Ref |  |
| Male | 0.75 | 0.26-2.15 | 0.587 |  |
| * Multivariable model was not performed due to low number of outcome events (n=14).  † BMI, Body mass index, based on weight and height before pregnancy, self-reported at first antenatal visit.  ‡ SGA, Small for gestational age infant. Defined as birth weight at least 2 SD below the mean for gestational age.  § Self-reported in a questionnaire.  FINNPEC, the Finnish Genetics of Pre-eclampsia Consortium; HR, hazard ratio; CI, confidence interval; wg, weeks of gestation; Ref, reference. | | | | |

| **Table S6.** Baseline characteristics of women with a diagnosis of severe or moderate preeclampsia in the FINNPEC Study. | | | |  |
| --- | --- | --- | --- | --- |
| Characteristic | Severe preeclampsia  (n=753) | Moderate preeclampsia (n=386) | *P*-value | |
| Maternal age at delivery, years, Mean (SD) | 30.7 (5.4) | 29.0 (5.1) | **<0.001** | |
| BMI* before pregnancy, kg/m^2^, Median (IQR) | 23.4 (21.3–26.9) | 23.6 (21.2–26.8) | 0.792 | |
| Primiparous, n (%) | 611 (81.1) | 300 (77.7) | 0.172 | |
| Gestational age at first antenatal visit, weeks, Mean (SD) | 9.0 (1.5) | 8.9 (1.4) | 0.273 | |
| Preterm delivery (<37^0/7^ wg), n (%) | 336 (44.6) | 44 (11.4) | **<0.001** | |
| Comorbidities |  |  |  | |
| Gestational diabetes, n (%) | 93 (12.4) | 43 (11.1) | 0.551 | |
| Pregestational type 1 or 2 diabetes, n (%) | 20 (2.7) | 10 (2.6) | 1.000 | |
| Placental insufficiency, n (%) | 83 (11.0) | 18 (4.7) | **<0.001** | |
| SGA†, n (%) | 203 (27.0) | 43 (11.1) | **<0.001** | |
| Preeclampsia characteristics |  |  |  | |
| Preeclampsia duration, days, Median (IQR) | 10 (5–16) | 7 (4–11) | **<0.001** | |
| Early-onset preeclampsia (delivery <34^0/7^ wg), n (%) | 167 (22.2) | 10 (2.6) | **<0.001** | |
| Early-onset preeclampsia (diagnosis <34^0/7^ wg), n (%) | 262 (34.8) | 22 (5.7) | **<0.001** | |
| Preeclampsia in mother’s first-degree relatives‡, n (%) | 50 (6.6) | 37 (9.6) | 0.076 | |
| SBP at first antenatal visit, mmHg, Mean (SD) | 121.7 (10.4) | 120.4 (10.3) | **0.044** | |
| DBP at first antenatal visit, mmHg, Mean (SD) | 75.7 (7.9) | 73.9 (8.5) | **<0.001** | |
| Highest SBP during pregnancy, mmHg, Mean (SD) | 170.9 (16.0) | 150.4 (11.3) | **<0.001** | |
| Highest DBP during pregnancy, mmHg, Mean (SD) | 111.2 (7.6) | 101.8 (6.3) | **<0.001** | |

| Maximum proteinuria, g/24 h, Median (IQR) | 4.4 (1.9–7.2) | 1.5 (0.8–2.6) | **<0.001** |
| --- | --- | --- | --- |
| Eclampsia seizures, n (%) | 11 (1.5) | 0 (0.0) | N/A |
| HELLP§, n (%) | 71 (9.4) | 10 (2.6) | **<0.001** |
| Smoking, n (%) |  |  | **0.009** |
| Never | 180 (23.9) | 88 (22.8) |  |
| Ever | 212 (28.2) | 142 (36.8) |  |
| Data missing | 361 (47.9) | 156 (40.4) |  |
| Sex of the child, n (%) |  |  | 0.052 |
| Female | 408 (54.3) | 187 (48.2) |  |
| Male | 344 (45.7) | 201 (51.8) |  |
| Composite cardiovascular disease during follow-up, n (%) | 43 (5.7) | 14 (3.6) | 0.151 |
| * BMI, Body mass index, based on weight and height before pregnancy, self-reported at first antenatal visit.  † SGA, Small for gestational age infant. Defined as birth weight at least 2 SD below the mean for gestational age.  ‡ Self-reported in a questionnaire.  § HELLP, Hemolysis, elevated liver enzymes, low platelet count.  FINNPEC, the Finnish Genetics of Pre-eclampsia Consortium; SD, standard deviation; IQR, interquartile range. | | | |

| **Table S7.** Subgroup analysis: risk factors for maternal cardiovascular diseases in women with history of severe preeclampsia in the FinnGen study (n=834). | | | | | | | | |
| --- | --- | --- | --- | --- | --- | --- | --- | --- |
| Variable | Univariate | | |  | Multivariable* | | | |
|  | HR | 95% CI | *P*-value | |  | HR | 95% CI | *P*-value |
| Preeclampsia duration, hazard per day | 1.00 | 0.99–1.01 | 0.908 | |  | Not entered |  |  |
| Early-onset preeclampsia (delivery <34^0/7^ wg) | 1.38 | 1.01–1.89 | **0.041** | |  | Not entered |  |  |
| Early-onset preeclampsia (diagnosis <34^0/7^ wg) | 1.34 | 1.00–1.78 | **0.049** | |  | Not entered |  |  |
| Maternal age at delivery | 0.95 | 0.92–0.98 | **<0.001** | |  | 0.95 | 0.92–0.97 | **<0.001** |
| Primiparity | 1.30 | 0.94–1.80 | 0.113 | |  | Not entered |  |  |
| Preterm delivery (<37^0/7^ wg) | 1.23 | 0.91–1.65 | 0.174 | |  | 1.57 | 1.14–2.15 | **0.005** |
| Gestational diabetes | 1.23 | 0.73–2.09 | 0.435 | |  | 1.95 | 1.13–3.39 | **0.017** |
| Pregestational type 1 diabetes | 4.79 | 3.13–7.35 | **<0.001** | |  | 4.80 | 3.10–7.44 | **<0.001** |
| Pregestational type 2 diabetes | 7.80 | 1.93–31.56 | **0.004** | |  | 16.16 | 3.89–67.16 | **<0.001** |
| SGA† | 0.93 | 0.57–1.52 | 0.775 | |  | Not entered |  |  |
| Sex of the child |  |  |  | |  |  |  |  |
| Female | Ref | Ref | Ref | |  | Ref | Ref | Ref |
| Male | 1.31 | 0.98–1.74 | 0.071 | |  | 1.37 | 1.02–1.83 | **0.036** |
| * Backward and forward selection resulted in identical models.  † SGA, Small for gestational age infant. Defined as ICD-9 diagnosis 656.5 or ICD-10 diagnosis O36.5.  HR, hazard ratio; CI, confidence interval; wg, weeks of gestation. | | | | | | | | |

| **Table S8.** Subgroup analysis: risk factors for maternal cardiovascular diseases in women with history of moderate preeclampsia in the FinnGen study (n=2769). | | | | | | | | | | | | | | | | |
| --- | --- | --- | --- | --- | --- | --- | --- | --- | --- | --- | --- | --- | --- | --- | --- | --- |
| Variable | | Univariate | | |  | Multivariable, backward selection | | | | |  | | Multivariable, forward selection | | | |
|  | | HR | 95% CI | *P*-value | | |  | HR | 95% CI | *P*-value | |  | | HR | 95% CI | *P*-value |
| Preeclampsia duration, hazard per day | | 1.01 | 1.01–1.02 | **<0.001** | | |  | 1.01 | 1.00–1.02 | **<0.001** | |  | | 1.01 | 1.00–1.02 | **0.002** |
| Early-onset preeclampsia (delivery <34^0/7^ wg) | | 1.21 | 0.75–1.96 | 0.439 | | |  | 1.66 | 1.02–2.71 | **0.042** | |  | | Not entered |  |  |
| Early-onset preeclampsia (diagnosis <34^0/7^ wg) | | 1.31 | 1.01–1.69 | **0.039** | | |  | Not entered |  |  | |  | | Not entered |  |  |
| Maternal age at delivery* | | 0.98 | 0.98–0.99 | **<0.001** | | |  | 0.98 | 0.97–0.98 | **<0.001** | |  | | 0.99 | 0.99–1.00 | 0.101 |
| Primiparity | | 0.95 | 0.79–1.13 | 0.566 | | |  | 0.74 | 0.61–0.90 | **0.002** | |  | | Not entered |  |  |
| Preterm delivery (<37^0/7^ wg) | | 0.94 | 0.72–1.22 | 0.647 | | |  | Not entered |  |  | |  | | Not entered |  |  |
| Gestational diabetes | | 2.08 | 1.65–2.61 | **<0.001** | | |  | 2.70 | 2.13–3.42 | **<0.001** | |  | | 2.25 | 1.79–2.84 | **<0.001** |
| Pregestational type 1 diabetes | | 2.97 | 1.98–4.44 | **<0.001** | | |  | 3.45 | 2.29–5.19 | **<0.001** | |  | | 3.23 | 2.15–4.85 | **<0.001** |
| Pregestational type 2 diabetes | | 5.56 | 2.76–11.19 | **<0.001** | | |  | 5.26 | 2.59–10.68 | **<0.001** | |  | | 5.40 | 2.65–11.00 | **<0.001** |
| SGA† | | 0.72 | 0.46–1.11 | 0.140 | | |  | Not entered |  |  | |  | | 0.73 | 0.47–1.14 | 0.165 |
| Sex of the child | |  |  |  | | |  | Not entered |  |  | |  | | Not entered |  |  |
| Female | | Ref | Ref | Ref | | |  |  |  |  | |  | |  |  |  |
| Male | | 0.93 | 0.78–1.10 | 0.388 | | |  |  |  |  | |  | |  |  |  |
|  | * Included as time-dependent due to violation of proportional hazards assumption.  † SGA, Small for gestational age infant. Defined as ICD-9 diagnosis 656.5 or ICD-10 diagnosis O36.5.  HR, hazard ratio; CI, confidence interval; wg, weeks of gestation. | | | | | | | | | | | | | | | |

| **Table S9.** Baseline characteristics of women with a diagnosis of severe or moderate preeclampsia in the FinnGen study. | | | |  |
| --- | --- | --- | --- | --- |
| Characteristic | Severe preeclampsia  (n=834) | Moderate preeclampsia (n=2769) | *P*-value | |
| Maternal age at delivery, years, Mean (SD) | 30.2 (5.6) | 29.8 (5.5) | 0.096 | |
| Primiparous, n (%) | 607 (72.8) | 1840 (66.4) | **<0.001** | |
| Preterm delivery (<37^0/7^ wg), n (%) | 462 (55.4) | 374 (13.5) | **<0.001** | |
| Comorbidities |  |  |  | |
| Gestational diabetes, n (%) | 69 (8.3) | 317 (11.4) | **0.011** | |
| Pregestational type 1 or 2 diabetes, n (%) | 50 (6.0) | 100 (3.6) | **0.003** | |
| SGA*, n (%) | 93 (11.2) | 155 (5.6) | **<0.001** | |
| Preeclampsia characteristics |  |  |  | |
| Preeclampsia duration, days, Median (IQR) | 7 (3–14) | 6 (3–13) | 0.086 | |
| Early-onset preeclampsia (delivery <34^0/7^ wg), n (%) | 215 (25.8) | 77 (2.8) | **<0.001** | |
| Early-onset preeclampsia (diagnosis <34^0/7^ wg), n (%) | 364 (43.6) | 302 (10.9) | **<0.001** | |
| Eclampsia seizures, n (%) | 41 (4.9) | 0 (0.0) | N/A | |
| Sex of the child, n (%) |  |  | 0.239 | |
| Female | 442 (53.0) | 1401 (50.6) |  | |
| Male | 392 (47.0) | 1368 (49.4) |  | |
| Composite cardiovascular disease during follow-up, n (%) | 185 (22.2) | 504 (18.2) | **0.012** | |
| * SGA, Small for gestational age infant. Defined as ICD-9 diagnosis 656.5 or ICD-10 diagnosis O36.5.  SD, standard deviation; IQR, interquartile range. | | | | |

**Collaborative Authorship Groups**

**The Finnish Genetics of Pre-eclampsia Consortium (FINNPEC) Core Investigator Group**

Hannele Laivuori Principal investigator. Medical and [Clinical Genetics](https://www.sciencedirect.com/topics/medicine-and-dentistry/clinical-genetics), University of Helsinki, and Helsinki University Hospital. Institute for Molecular Medicine Finland, Helsinki Institute of Life Science, University of Helsinki. Department of [Obstetrics](https://www.sciencedirect.com/topics/medicine-and-dentistry/obstetrics) and [Gynecology](https://www.sciencedirect.com/topics/medicine-and-dentistry/gynecology), Tampere University Hospital, The Wellbeing services county of Pirkanmaa, Center for Child, Adolescent and Maternal Health Research, Faculty of Medicine, and [Health Technology](https://www.sciencedirect.com/topics/medicine-and-dentistry/health-technology), Tampere University.

Seppo Heinonen Obstetrics and Gynecology, University of Helsinki and Helsinki University Hospital.

Eero Kajantie PEDEGO Research Unit, [Medical Research](https://www.sciencedirect.com/topics/medicine-and-dentistry/medical-research) Center Oulu, Oulu University Hospital and University of Oulu. [Public Health](https://www.sciencedirect.com/topics/medicine-and-dentistry/public-health) Promotion Unit, National Institute for Health and Welfare, Helsinki, and Oulu. Children’s hospital, University of Helsinki and Helsinki University Hospital. Department of Clinical and Molecular Medicine, Norwegian University of Health and Technology, Trondheim, Norway.

Juha Kere Department of Biosciences and Nutrition, Karolinska Institutet, Huddinge, Sweden. Folkhalsan Institute of Genetics and Molecular [Neurology](https://www.sciencedirect.com/topics/medicine-and-dentistry/neurology) Research Program, University of Helsinki, School of Basic & Medical Biosciences, King’s College London, London, England.

Katja Kivinen Institute for Molecular Medicine Finland, Helsinki Institute of Life Science, University of Helsinki.

Anneli Pouta Department of Government Services, National Institute for Health and Welfare, Helsinki.

**Contributors of FinnGen**

**Steering Committee**

Aarno Palotie Institute for Molecular Medicine Finland (FIMM), HiLIFE, University of Helsinki, Helsinki, Finland; Broad Institute of MIT and Harvard; Massachusetts General Hospital

Mark Daly Institute for Molecular Medicine Finland (FIMM), HiLIFE, University of Helsinki, Helsinki, Finland; Broad Institute of MIT and Harvard; Massachusetts General Hospital

**Pharmaceutical companies**

Bridget Riley-Gills Abbvie, Chicago, IL, United States

Howard Jacob Abbvie, Chicago, IL, United States

Coralie Viollet Astra Zeneca, Cambridge, United Kingdom

Slavé Petrovski Astra Zeneca, Cambridge, United Kingdom

Alix Berton Bayer AG, Leverkusen, Germany

Santha Ramakrishnan Bayer AG, Leverkusen, Germany

Ellen Tsai Biogen, Cambridge, MA, United States

Zhihao Ding Boehringer Ingelheim, Ingelheim am Rhein, Germany

Emily Holzinger Bristol Myers Squibb, New York, NY, United States

Robert Plenge Bristol Myers Squibb, New York, NY, United States

Joseph Maranville Bristol Myers Squibb, New York, NY, United States

Mark McCarthy Genentech, San Francisco, CA, United States

Rion Pendergrass Genentech, San Francisco, CA, United States

Jonathan Davitte GlaxoSmithKline, Collegeville, PA, United States

Chia-Yen Chen Merck, Kenilworth, NJ, United States

Melis Atalar Aksit Pfizer, New York, NY, United States

Anna Vlahiotis Pfizer, New York, NY, United States

Katherine Klinger Translational Sciences, Sanofi R&D, Framingham, MA, USA

Clement Chatelain Translational Sciences, Sanofi R&D, Framingham, MA, USA

Jorg Blankenstein Translational Sciences, Sanofi R&D, Framingham, MA, USA

Karol Estrada Maze Therapeutics, San Francisco, CA, United States

Robert Graham Maze Therapeutics, San Francisco, CA, United States

Dawn Waterworth Johnson & Johnson Innovative Medicine, Spring House, PA, United States

Chris O´Donnell Novartis Institutes for BioMedical Research, Cambridge, MA, United States

Nicole Renaud Novartis Institutes for BioMedical Research, Cambridge, MA, United States

**University of Helsinki & Biobanks**

Tomi P. Mäkelä HiLIFE, University of Helsinki, Finland, Finland

Jaakko Kaprio Institute for Molecular Medicine Finland (FIMM), HiLIFE, University of Helsinki, Helsinki, Finland

Minna Ruddock Arctic biobank / University of Oulu

Lila Kallio Auria Biobank / University of Turku / Wellbeing Services County of Southwest Finland, Turku, Finland

Antti Hakanen Auria Biobank / University of Turku / Wellbeing Services County of Southwest Finland, Turku, Finland

Terhi Kilpi THL Biobank / Finnish Institute for Health and Welfare (THL), Helsinki, Finland

Markus Perola THL Biobank / Finnish Institute for Health and Welfare (THL), Helsinki, Finland

Jukka Partanen Finnish Red Cross Blood Service / Finnish Hematology Registry and Clinical Biobank, Helsinki, Finland

Taneli Raivio Helsinki Biobank / Helsinki University and Hospital District of Helsinki and Uusimaa, Helsinki

Eero Punkka Helsinki Biobank / Helsinki University and Hospital District of Helsinki and Uusimaa, Helsinki

Teija Kekonen Northern Finland Biobank Borealis / University of Oulu / Wellbeing services county of North Ostrobothnia, Oulu, Finland

Raisa Serpi Northern Finland Biobank Borealis / University of Oulu / Wellbeing services county of North Ostrobothnia, Oulu, Finland

Kati Kristiansson Finnish Clinical Biobank Tampere / University of Tampere / Wellbeing Services County of Pirkanmaa, Tampere, Finland

Sanna Siltanen Finnish Clinical Biobank Tampere / University of Tampere / Wellbeing Services County of Pirkanmaa, Tampere, Finland

Veli-Matti Kosma Biobank of Eastern Finland / University of Eastern Finland / Wellbeing services county of North Savo, Kuopio, Finland

Arto Mannermaa Biobank of Eastern Finland / University of Eastern Finland / Wellbeing services county of North Savo, Kuopio, Finland

Jari Laukkanen Central Finland Biobank / University of Jyväskylä / Wellbeing Services County of Central Finland, Jyväskylä, Finland

Tiina Jokela Central Finland Biobank / University of Jyväskylä / Wellbeing Services County of Central Finland, Jyväskylä, Finland

Mervi Ahlroth Finnish Biobank Cooperative - FINBB

Johanna Mäkelä Finnish Biobank Cooperative – FINBB

**Other Experts/ Non-Voting Members**

Outi Tuovila Business Finland, Helsinki, Finland

**Scientific Committee**

**Pharmaceutical companies**

Jeffrey Waring Abbvie, Chicago, IL, United States

Bridget Riley-Gillis Abbvie, Chicago, IL, United States

Fedik Rahimov Abbvie, Chicago, IL, United States

Ioanna Tachmazidou Astra Zeneca, Cambridge, United Kingdom

Slavé Petrovski Astra Zeneca, Cambridge, United Kingdom

Alix Berton Bayer AG, Leverkusen, Germany

Santha Ramakrishnan Bayer AG, Leverkusen, Germany

Ellen Tsai Biogen, Cambridge, MA, United States

Zhihao Ding Boehringer Ingelheim, Ingelheim am Rhein, Germany

Marc Jung Boehringer Ingelheim, Ingelheim am Rhein, Germany

Hanati Tuoken Boehringer Ingelheim, Ingelheim am Rhein, Germany

Shameek Biswas Bristol Myers Squibb, New York, NY, United States

Benjamin Sun Bristol Myers Squibb, New York, NY, United States

Rion Pendergrass Genentech, San Francisco, CA, United States

Jonathan Davitte GlaxoSmithKline, Collegeville, PA, United States

Neha Raghavan Merck, Kenilworth, NJ, United States

Jae-Hoon Sul Merck, Kenilworth, NJ, United States

Melis Atalar Aksit Pfizer, New York, NY, United States

Xinli Hu Pfizer, New York, NY, United States

Katherine Klinger Translational Sciences, Sanofi R&D, Framingham, MA, USA

Robert Graham Maze Therapeutics, San Francisco, CA, United States

Dawn Waterworth Johnson & Johnson Innovative Medicine, Spring House, PA, United States

Nicole Renaud Novartis Institutes for BioMedical Research, Cambridge, MA, United States

Ma´en Obeidat Novartis Institutes for BioMedical Research, Cambridge, MA, United States

Jonathan Chung Novartis Institutes for BioMedical Research, Cambridge, MA, United States

Jonas Zierer Novartis Institutes for BioMedical Research, Cambridge, MA, United States

Mari Niemi Novartis Institutes for BioMedical Research, Cambridge, MA, United States

**University of Helsinki & Biobanks**

Samuli Ripatti Institute for Molecular Medicine Finland (FIMM), HiLIFE, University of Helsinki, Helsinki, Finland

Johanna Schleutker Auria Biobank / University of Turku / Wellbeing Services County of Southwest Finland, Turku, Finland

Markus Perola THL Biobank / Finnish Institute for Health and Welfare (THL), Helsinki, Finland

Tiina Wahlfors THL Biobank / Finnish Institute for Health and Welfare (THL), Helsinki, Finland

Mikko Arvas Finnish Red Cross Blood Service / Finnish Hematology Registry and Clinical Biobank, Helsinki, Finland

Olli Carpén Helsinki Biobank / Helsinki University and Hospital District of Helsinki and Uusimaa, Helsinki

Reetta Hinttala Northern Finland Biobank Borealis / University of Oulu / Wellbeing services county of North Ostrobothnia, Oulu, Finland

Johannes Kettunen Northern Finland Biobank Borealis / University of Oulu / Wellbeing services county of North Ostrobothnia, Oulu, Finland

Arto Mannermaa Biobank of Eastern Finland / University of Eastern Finland / Wellbeing services county of North Savo, Kuopio, Finland

Katriina Aalto-Setälä Faculty of Medicine and Health Technology, Tampere University, Tampere, Finland

Mika Kähönen Finnish Clinical Biobank Tampere / University of Tampere / Wellbeing Services County of Pirkanmaa, Tampere, Finland

Jari Laukkanen Central Finland Biobank / University of Jyväskylä / Wellbeing Services County of Central Finland, Jyväskylä, Finland

Johanna Mäkelä FINBB - Finnish biobank cooperative

**Clinical Group / Task Force**

Hanna Kujala Biobank of Eastern Finland / University of Eastern Finland / Wellbeing services county of North Savo, Kuopio, Finland

Triin Laisk Estonian biobank, Tartu, Estonia

Natalia Pujol Estonian biobank, Tartu, Estonia

Mika Kähönen Finnish Clinical Biobank Tampere / University of Tampere / Wellbeing Services County of Pirkanmaa, Tampere, Finland

Veikko Salomaa Finnish Institute for Health and Welfare (THL), Helsinki, Finland

Jaana Suvisaari Finnish Institute for Health and Welfare (THL), Helsinki, Finland

Satu Koskela Finnish Red Cross Blood Service / Finnish Hematology Registry and Clinical Biobank, Helsinki, Finland

Jouni Lauronen Finnish Red Cross Blood Service / Finnish Hematology Registry and Clinical Biobank, Helsinki, Finland

Kristiina Aittomäki Helsinki University Central Hospital, Helsinki, Finland

Pirkko Pussinen Helsinki University Hospital and University of Helsinki, Helsinki / University of Eastern Finland. Kuopio, Finland

Tuomo Meretoja Helsinki University Hospital and University of Helsinki, Helsinki, Finland

Heikki Joensuu Helsinki University Hospital and University of Helsinki, Helsinki, Finland

Peeter Karihtala Helsinki University Hospital and University of Helsinki, Helsinki, Finland

Emma Juuri Helsinki University Hospital and University of Helsinki, Helsinki, Finland

Aino Salminen Helsinki University Hospital and University of Helsinki, Helsinki, Finland

Tuula Salo Helsinki University Hospital and University of Helsinki, Helsinki, Finland

David Rice Helsinki University Hospital and University of Helsinki, Helsinki, Finland

Pekka Nieminen Helsinki University Hospital and University of Helsinki, Helsinki, Finland

Ulla Palotie Helsinki University Hospital and University of Helsinki, Helsinki, Finland

Fredrik Åberg Helsinki University Hospital and University of Helsinki, Helsinki, Finland

Daniel Gordin Helsinki University Hospital and University of Helsinki, Helsinki, Finland

Patrik Finne Helsinki University Hospital and University of Helsinki, Helsinki, Finland

Joni A Turunen Helsinki University Hospital and University of Helsinki, Helsinki, Finland; Folkhälsan Research Center, Helsinki, Finland

Minna Raivio Hospital District of Helsinki and Uusimaa, Helsinki, Finland

Pentti Tienari Hospital District of Helsinki and Uusimaa, Helsinki, Finland

Martti Färkkilä Hospital District of Helsinki and Uusimaa, Helsinki, Finland

Jukka Koskela Hospital District of Helsinki and Uusimaa, Helsinki, Finland

Sampsa Pikkarainen Hospital District of Helsinki and Uusimaa, Helsinki, Finland

Kari Eklund Hospital District of Helsinki and Uusimaa, Helsinki, Finland

Paula Kauppi Hospital District of Helsinki and Uusimaa, Helsinki, Finland

Daniel Gordin Hospital District of Helsinki and Uusimaa, Helsinki, Finland

Juha Sinisalo Hospital District of Helsinki and Uusimaa, Helsinki, Finland

Marja-Riitta Taskinen Hospital District of Helsinki and Uusimaa, Helsinki, Finland

Tiinamaija Tuomi Hospital District of Helsinki and Uusimaa, Helsinki, Finland

Timo Hiltunen Hospital District of Helsinki and Uusimaa, Helsinki, Finland

Johanna Mattson Hospital District of Helsinki and Uusimaa, Helsinki, Finland

Eveliina Salminen Hospital District of Helsinki and Uusimaa, Helsinki, Finland

Terhi Ollila Hospital District of Helsinki and Uusimaa, Helsinki, Finland

Katariina Hannula-Jouppi Hospital District of Helsinki and Uusimaa, Helsinki, Finland

Oskari Heikinheimo Hospital District of Helsinki and Uusimaa, Helsinki, Finland

Ilkka Kalliala Hospital District of Helsinki and Uusimaa, Helsinki, Finland

Lauri Aaltonen Hospital District of Helsinki and Uusimaa, Helsinki, Finland

Erkki Isometsä Hospital District of Helsinki and Uusimaa, Helsinki, Finland

Antti Aarnisalo Hospital District of Helsinki and Uusimaa, Helsinki, Finland

Ilkka Immonen Hospital District of Helsinki and Uusimaa, Helsinki, Finland

Salla Ranta Hospital District of Helsinki and Uusimaa, Helsinki, Finland

Filip Scheperjans Hospital District of Helsinki and Uusimaa, Helsinki, Finland

Felix Vaura Institute for Molecular Medicine Finland (FIMM), HiLIFE, University of Helsinki, Helsinki, Finland

Nina Mars Institute for Molecular Medicine Finland (FIMM), HiLIFE, University of Helsinki, Helsinki, Finland

Esa Pitkänen Institute for Molecular Medicine Finland (FIMM), HiLIFE, University of Helsinki, Helsinki, Finland

Hannele Laivuori Institute for Molecular Medicine Finland (FIMM), HiLIFE, University of Helsinki, Helsinki, Finland

Katja Kivinen Institute for Molecular Medicine Finland (FIMM), HiLIFE, University of Helsinki, Helsinki, Finland

Elisabeth Widen Institute for Molecular Medicine Finland (FIMM), HiLIFE, University of Helsinki, Helsinki, Finland

Taru Tukiainen Institute for Molecular Medicine Finland (FIMM), HiLIFE, University of Helsinki, Helsinki, Finland

Hanna Ollila Institute for Molecular Medicine Finland (FIMM), HiLIFE, University of Helsinki, Helsinki, Finland

Elmo Saarentaus Institute for Molecular Medicine Finland (FIMM), HiLIFE, University of Helsinki, Helsinki, Finland

Anne Kerola Institute for Molecular Medicine Finland (FIMM), HiLIFE, University of Helsinki, Helsinki, Finland

Eero Vuoksimaa Institute for Molecular Medicine Finland (FIMM), HiLIFE, University of Helsinki, Helsinki, Finland

Joni Lindbohm Institute for Molecular Medicine Finland (FIMM), HiLIFE, University of Helsinki, Helsinki, Finland

Zhiyu Yang Institute for Molecular Medicine Finland (FIMM), HiLIFE, University of Helsinki, Helsinki, Finland

Matthew Sampson Institute for Molecular Medicine Finland (FIMM), HiLIFE, University of Helsinki, Helsinki, Finland; Broad Institute & Harvard Medical School, Cambridge, United States

Adrian Banerji Institute for Molecular Medicine Finland (FIMM), HiLIFE, University of Helsinki, Helsinki, Finland; Broad Institute & Harvard Medical School, Cambridge, United States

Michelle McNulty Institute for Molecular Medicine Finland (FIMM), HiLIFE, University of Helsinki, Helsinki, Finland; Broad Institute & Harvard Medical School, Cambridge, United States

Aoxing Liu Institute for Molecular Medicine Finland (FIMM), HiLIFE, University of Helsinki, Helsinki, Finland; Broad Institute, Cambridge, MA, United States

Joel Rämö Institute for Molecular Medicine Finland (FIMM), HiLIFE, University of Helsinki, Helsinki, Finland; Broad Institute, Cambridge, MA, United States

Austin Argentieri Institute for Molecular Medicine Finland (FIMM), HiLIFE, University of Helsinki, Helsinki, Finland; Broad Institute, Cambridge, MA, United States

Amanda Elliott Institute for Molecular Medicine Finland (FIMM), HiLIFE, University of Helsinki, Helsinki, Finland; Broad Institute, Cambridge, MA, USA and Massachusetts General Hospital, Boston, MA, USA

Elisa Rahikkala Northern Ostrobothnia Hospital District, Oulu, Finland

Kirsi Sipilä Oulu University Hospital and University of Oulu, Oulu, Finland

Valtteri Julkunen University of Eastern Finland and Kuopio University Hospital, Kuopio, Finland

Ville Leinonen University of Eastern Finland and Kuopio University Hospital, Kuopio, Finland

Sanna Toppila-Salmi University of Eastern Finland and Kuopio University Hospital, Kuopio, Finland; Helsinki University Hospital and University of Helsinki, Finland

Mikko Hiltunen University of Eastern Finland, Kuopio, Finland

Eino Solje University of Eastern Finland, Kuopio, Finland

Hannu Kankaanranta University of Gothenburg, Gothenburg, Sweden/ Seinäjoki Central Hospital, Seinäjoki, Finland/ Tampere University, Tampere, Finland

Antti Mäkitie University of Helsinki and Helsinki University Hospital, Helsinki, Finland

Iiris Hovatta University of Helsinki, Helsinki, Finland

Niko Välimäki University of Helsinki, Helsinki, Finland

Minttu Marttila University of Helsinki, Helsinki, Finland

Anne Portaankorva University of Helsinki, Helsinki, Finland

Eija Laakkonen University of Jyväskylä, Jyväskylä, Finland

Heidi Silven University of Oulu, Oulu, Finland

Eeva Sliz University of Oulu, Oulu, Finland

Riikka Arffman University of Oulu, Oulu, Finland

Susanna Savukoski University of Oulu, Oulu, Finland

Riitta Kaarteenaho University of Oulu, Oulu, Finland

Jaakko Tyrmi University of Oulu, Oulu, Finland / University of Tampere, Tampere, Finland

Laura Kuusalo University of Turku, Turku, Finland

Laura Pirilä University of Turku, Turku, Finland

Tapio Hellman University of Turku, Turku, Finland

Matti Vuori University of Turku, Turku, Finland

Teemu Niiranen University of Turku, Turku, Finland; Finnish Institute for Health and Welfare (THL), Helsinki, Finland

Timo Blomster Wellbeing services county of North Ostrobothnia, Oulu, Finland

Johanna Huhtakangas Wellbeing services county of North Ostrobothnia, Oulu, Finland

Terttu Harju Wellbeing services county of North Ostrobothnia, Oulu, Finland

Kaisa Tasanen Wellbeing services county of North Ostrobothnia, Oulu, Finland

Laura Huilaja Wellbeing services county of North Ostrobothnia, Oulu, Finland

Vuokko Anttonen Wellbeing services county of North Ostrobothnia, Oulu, Finland

Marja Vääräsmäki Wellbeing services county of North Ostrobothnia, Oulu, Finland

Outi Uimari Wellbeing services county of North Ostrobothnia, Oulu, Finland

Laure Morin-Papunen Wellbeing services county of North Ostrobothnia, Oulu, Finland

Maarit Niinimäki Wellbeing services county of North Ostrobothnia, Oulu, Finland

Terhi Piltonen Wellbeing services county of North Ostrobothnia, Oulu, Finland

Reetta Kälviäinen Wellbeing services county of North Savo, Kuopio, Finland

Valtteri Julkunen Wellbeing services county of North Savo, Kuopio, Finland

Hilkka Soininen Wellbeing services county of North Savo, Kuopio, Finland

Mikko Kiviniemi Wellbeing services county of North Savo, Kuopio, Finland

Oili Kaipiainen-Seppänen Wellbeing services county of North Savo, Kuopio, Finland

Margit Pelkonen Wellbeing services county of North Savo, Kuopio, Finland

Päivi Auvinen Wellbeing services county of North Savo, Kuopio, Finland

Maria Siponen Wellbeing services county of North Savo, Kuopio, Finland

Liisa Suominen Wellbeing services county of North Savo, Kuopio, Finland

Päivi Mäntylä Wellbeing services county of North Savo, Kuopio, Finland

Kai Kaarniranta Wellbeing services county of North Savo, Kuopio, Finland; University of Lodz, Lodz, Poland

Jukka Peltola Wellbeing Services County of Pirkanmaa, Tampere, Finland

Airi Jussila Wellbeing Services County of Pirkanmaa, Tampere, Finland

Katri Kaukinen Wellbeing Services County of Pirkanmaa, Tampere, Finland

Pia Isomäki Wellbeing Services County of Pirkanmaa, Tampere, Finland

Jussi Hernesniemi Wellbeing Services County of Pirkanmaa, Tampere, Finland

Annika Auranen Wellbeing Services County of Pirkanmaa, Tampere, Finland

Hannu Uusitalo Wellbeing Services County of Pirkanmaa, Tampere, Finland

Teea Salmi Wellbeing Services County of Pirkanmaa, Tampere, Finland

Venla Kurra Wellbeing Services County of Pirkanmaa, Tampere, Finland

Laura Kotaniemi-Talonen Wellbeing Services County of Pirkanmaa, Tampere, Finland

Argyro Bizaki-Vallaskangas Wellbeing Services County of Pirkanmaa, Tampere, Finland

Juha Rinne Wellbeing Services County of Southwest Finland, Turku, Finland

Roosa Kallionpää Wellbeing Services County of Southwest Finland, Turku, Finland

Markku Voutilainen Wellbeing Services County of Southwest Finland, Turku, Finland

Antti Palomäki Wellbeing Services County of Southwest Finland, Turku, Finland

Laura Pirilä Wellbeing Services County of Southwest Finland, Turku, Finland

Riitta Lahesmaa Wellbeing Services County of Southwest Finland, Turku, Finland

Kaj Metsärinne Wellbeing Services County of Southwest Finland, Turku, Finland

Jenni Aittokallio Wellbeing Services County of Southwest Finland, Turku, Finland

Klaus Elenius Wellbeing Services County of Southwest Finland, Turku, Finland

Sirkku Peltonen Wellbeing Services County of Southwest Finland, Turku, Finland

Leena Koulu Wellbeing Services County of Southwest Finland, Turku, Finland

Ulvi Gursoy Wellbeing Services County of Southwest Finland, Turku, Finland

Varpu Jokimaa Wellbeing Services County of Southwest Finland, Turku, Finland

Tytti Willberg Wellbeing Services County of Southwest Finland, Turku, Finland

Adam Ziemann Abbvie, Chicago, IL, United States

Nizar Smaoui Abbvie, Chicago, IL, United States

Anne Lehtonen Abbvie, Chicago, IL, United States

Apinya Lertratanakul Abbvie, Chicago, IL, United States

Relja Popovic Abbvie, Chicago, IL, United States

Mengzhen Liu Abbvie, Chicago, IL, United States

Anneke Den Hollander AbbVie, Chicago, IL, United States

Jan Freudenberg AbbVie, Chicago, IL, United States

Britney Milkovich AbbVie, Chicago, IL, United States

Andrew Blumenfeld AbbVie, Chicago, IL, United States

Tushar Kumar AbbVie, Chicago, IL, United States

Dirk Paul Astra Zeneca, Cambridge, United Kingdom

Bram Prins Astra Zeneca, Cambridge, United Kingdom

Eleanor Wheeler Astra Zeneca, Cambridge, United Kingdom

Kousik Kundu Astra Zeneca, Cambridge, United Kingdom

Santosh Atanur Astra Zeneca, Cambridge, United Kingdom

Andrew Lowe Astra Zeneca, Cambridge, United Kingdom

Thomas Spargo Astra Zeneca, Cambridge, United Kingdom

Oliver Burren Astra Zeneca, Cambridge, United Kingdom

Margarete Fabre AstraZeneca, Cambridge, United Kingdom

Fabio Baschiera Bayer AG, Leverkusen, Germany

Hans van Leeuwen Bayer AG, Leverkusen, Germany

Himanshu Manchanda Bayer AG, Leverkusen, Germany

Karl Heilbron Bayer AG, Leverkusen, Germany

Martin Rao Bayer AG, Leverkusen, Germany

Nicole Schmidt Bayer AG, Leverkusen, Germany

Samu Kurki Bayer AG, Leverkusen, Germany

Johanna Mielke Bayer AG, Leverkusen, Germany

Juho Immonen Bayer AG, Leverkusen, Germany

Thomas Battram Bayer AG, Leverkusen, Germany

Tobias Hogrebe Bayer AG, Leverkusen, Germany

Susan Eaton Biogen, Cambridge, MA, United States

Ketian Yu Biogen, Cambridge, MA, United States

Stephanie Loomis Biogen, Cambridge, MA, United States

Coro Paisan-Ruiz Biogen, Cambridge, MA, United States

Elke Markert Boehringer Ingelheim, Ingelheim am Rhein, Germany

Frank Li Boehringer Ingelheim, Ingelheim am Rhein, Germany

Yao Hu Boehringer Ingelheim, Ingelheim am Rhein, Germany

Christoph Ogris Boehringer Ingelheim, Ingelheim am Rhein, Germany

Eric Simon Boehringer Ingelheim, Ingelheim am Rhein, Germany

Julio Cesar Bolivar Lopez Boehringer Ingelheim, Ingelheim am Rhein, Germany

Monika Frysz Boehringer Ingelheim, Ingelheim am Rhein, Germany

Marla Hochfeld Bristol Myers Squibb, New York, NY, United States

Cara Carty Bristol Myers Squibb, New York, NY, United States

Michael Turchin Bristol Myers Squibb, New York, NY, United States

Neelakshi Jog Bristol Myers Squibb, New York, NY, United States

Corneliu Bodea Bristol Myers Squibb, New York, NY, United States

Janie Shelton Bristol Myers Squibb, New York, NY, United States

Chen Li Bristol Myers Squibb, New York, NY, United States

Kritika Singh Bristol Myers Squibb, New York, NY, United States

Peng Jiang Bristol Myers Squibb, New York, NY, United States

Stephanie Loomis Bristol Myers Squibb, New York, NY, United States

Elena Sanchez Bristol Myers Squibb, New York, NY, United States

Lilith Moss Bristol Myers Squibb, New York, NY, United States

Zijie Zhao Bristol Myers Squibb, New York, NY, United States

Anna Podgornaia Bristol Myers Squibb, New York, NY, United States

Natalie Bowers Genentech, San Francisco, CA, United States

Edmond Teng Genentech, San Francisco, CA, United States

Tim Lu Genentech, San Francisco, CA, United States

Hubert Chen Genentech, San Francisco, CA, United States

Jennifer Schutzman Genentech, San Francisco, CA, United States

Erich Strauss Genentech, San Francisco, CA, United States

Hao Chen Genentech, San Francisco, CA, United States

David Choy Genentech, San Francisco, CA, United States

Rion Pendergrass Genentech, San Francisco, CA, United States

Brian Yaspan Genentech, San Francisco, CA, United States

Cameron Adams Genentech, San Francisco, CA, United States

Mark McCarthy Genentech, San Francisco, CA, United States

Michael Rothenberg Genentech, San Francisco, CA, United States

Rion Pendergrass Genentech, San Francisco, CA, United States

Sergio Dellepiane Genentech, San Francisco, CA, United States

Anubha Mahajan Genentech, San Francisco, CA, United States

Michael Holmes Genentech, San Francisco, CA, United States

Anubha Mahajan Genentech, San Francisco, CA, United States

Diana Chang Genentech, San Francisco, CA, United States

Tushar Bhangale Genentech, San Francisco, CA, United States

Fanli Xu GlaxoSmithKline, Brentford, United Kingdom

Laura Addis GlaxoSmithKline, Brentford, United Kingdom

John Eicher GlaxoSmithKline, Brentford, United Kingdom

Linda McCarthy GlaxoSmithKline, Brentford, United Kingdom

Jorge Esparza Gordillo GlaxoSmithKline, Brentford, United Kingdom

Joanna Betts GlaxoSmithKline, Brentford, United Kingdom

Rajashree Mishra GlaxoSmithKline, Brentford, United Kingdom

Audrey Chu GlaxoSmithKline, Brentford, United Kingdom

Diptee Kulkarni GlaxoSmithKline, Brentford, United Kingdom

Janet Kumar GlaxoSmithKline, Collegeville, PA, United States

Charli Harlow GlaxoSmithKline, Collegeville, PA, United States

Lea Sarow-Blat GlaxoSmithKline, Collegeville, PA, United States

Diana L.Cousminer GlaxoSmithKline, Collegeville, PA, United States

Jagtar Nijjar GlaxoSmithKline, Collegeville, PA, United States

Jessica Chao GlaxoSmithKline, Collegeville, PA, United States

Michal Magid GlaxoSmithKline, Collegeville, PA, United States

Shashank Jariwala GlaxoSmithKline, Collegeville, PA, United States

Chris Floyd GlaxoSmithKline, Collegeville, PA, United States

Dan Swerdlow GlaxoSmithKline, Collegeville, PA, United States

Erding Hu GlaxoSmithKline, Collegeville, PA, United States

Prerak Desai GlaxoSmithKline, Collegeville, PA, United States

Stephen Haddad GlaxoSmithKline, Collegeville, PA, United States

Damien Croteau-Chonka GlaxoSmithKline, Collegeville, PA, United States

Billy Fahy GlaxoSmithKline, Collegeville, PA, United States

Paola Bronson GlaxoSmithKline, Collegeville, PA, United States

Kirsi Auro GlaxoSmithKline, Espoo, Finland

David Pulford GlaxoSmithKline, Stevenage, United Kingdom

Sauli Vuoti Janssen-Cilag Oy, Espoo, Finland

Dermot Reilly Johnson & Johnson Innovative Medicine, Boston, MA, United States

Karen He Johnson & Johnson Innovative Medicine, Spring House, PA, United States

Ekaterina Khramtsova Johnson & Johnson Innovative Medicine, Spring House, PA, United States

Amy Hart Johnson & Johnson Innovative Medicine, Spring House, PA, United States

Meijian Guan Johnson & Johnson Innovative Medicine, Spring House, PA, United States

Alessandro Porello Johnson & Johnson Innovative Medicine, Spring House, PA, United States

P. Dunnmon Johnson & Johnson Innovative Medicine, Spring House, PA, United States

Sara Gale Johnson & Johnson Innovative Medicine, Spring House, PA, United States

Brice Keyes Johnson & Johnson Innovative Medicine, Spring House, PA, United States

John Kwon Johnson & Johnson Innovative Medicine, Spring House, PA, United States

Jonathan Sherlock Johnson & Johnson Innovative Medicine, Spring House, PA, United States

Matt Loza Johnson & Johnson Innovative Medicine, Spring House, PA, United States

Chris Whelan Johnson & Johnson Innovative Medicine, Spring House, PA, United States

W Galpern Johnson & Johnson Innovative Medicine, Spring House, PA, United States

Yanfei Zhang Johnson & Johnson Innovative Medicine, Spring House, PA, United States

Mona Selej Johnson & Johnson Innovative Medicine, Spring House, PA, United States

Abolfazl Doostparast Torshizi Johnson & Johnson Innovative Medicine, Spring House, PA, United States

Qingqin S Li Johnson & Johnson Innovative Medicine, Titusville, NJ, United States

Sahar Mozzafari Maze Therapeutics, San Francisco, CA, United States

Christopher Deboever Maze Therapeutics, San Francisco, CA, United States

Jason Miller Merck, Kenilworth, NJ, United States

Fabiana Farias Merck, Kenilworth, NJ, United States

Andrey Loboda Merck, Kenilworth, NJ, United States

Jorge Del-aguila Merck, Kenilworth, NJ, United States

Elisabeth Vollmann Merck, Kenilworth, NJ, United States

Jozsef Karman Merck, Kenilworth, NJ, United States

Julie Fiore Merck, Kenilworth, NJ, United States

Rajesh Kamath Merck, Kenilworth, NJ, United States

Andrei Popescu Merck, Kenilworth, NJ, United States

Delphine Fagegaltier Merck, Kenilworth, NJ, United States

Travis Barr Merck, Kenilworth, NJ, United States

Aristide Merola Merck, Kenilworth, NJ, United States

Oliver Freeman Merck, Kenilworth, NJ, United States

Simonne Longerich Merck, Kenilworth, NJ, United States

Enrico Ferrero Novartis Institutes for BioMedical Research, Cambridge, MA, United States

Nikos Patsopoulos Novartis Institutes for BioMedical Research, Cambridge, MA, United States

Nancy Finkel Novartis Institutes for BioMedical Research, Cambridge, MA, United States

Sabina Pfister Novartis Institutes for BioMedical Research, Cambridge, MA, United States

Shola Richards Novartis Institutes for BioMedical Research, Cambridge, MA, United States

Katherine Mccauley Novartis Institutes for BioMedical Research, Cambridge, MA, United States

Xiaobo Xia Novartis Institutes for BioMedical Research, Cambridge, MA, United States

Mike Mendelson Novartis Institutes for BioMedical Research, Cambridge, MA, United States

Majd Mouded Novartis, Basel, Switzerland

Debby Ngo Novartis, Basel, Switzerland

Kirsi Kalpala Pfizer, New York, NY, United States

Melissa Miller Pfizer, New York, NY, United States

Nan Bing Pfizer, New York, NY, United States

Jaakko Parkkinen Pfizer, New York, NY, United States

Heli Lehtonen Pfizer, New York, NY, United States

Stefan McDonough Pfizer, New York, NY, United States

Ying Wu Pfizer, New York, NY, United States

Erin Macdonald-Dunlop Pfizer, New York, NY, United States

Jessica Chung Pfizer, New York, NY, United States

Michael McLean Pfizer, New York, NY, United States

Joshua Chiou Pfizer, New York, NY, United States

Hye In Kim Pfizer, New York, NY, United States

Sivakumar Pitchumani Pfizer, New York, NY, United States

Sumedha Jassal Pfizer, New York, NY, United States

Madhurima Saxena Pfizer, New York, NY, United States

Catherine O’Riordan Translational Sciences, Sanofi R&D, Framingham, MA, USA

Samuel Lessard Translational Sciences, Sanofi R&D, Framingham, MA, USA

Suzanne Jacobs Translational Sciences, Sanofi R&D, Framingham, MA, USA

Hamid Mattoo Translational Sciences, Sanofi R&D, Framingham, MA, USA

David Habiel Translational Sciences, Sanofi R&D, Framingham, MA, USA

Guanling Huan Translational Sciences, Sanofi R&D, Framingham, MA, USA

**Biobank directors**

Lila Kallio Auria Biobank / University of Turku / Wellbeing Services County of Southwest Finland, Turku, Finland

Tiina Wahlfors THL Biobank / Finnish Institute for Health and Welfare (THL), Helsinki, Finland

Jukka Partanen Finnish Red Cross Blood Service / Finnish Hematology Registry and Clinical Biobank, Helsinki, Finland

Eero Punkka Helsinki Biobank / Helsinki University and Hospital District of Helsinki and Uusimaa, Helsinki

Raisa Serpi Northern Finland Biobank Borealis / University of Oulu / Wellbeing services county of North Ostrobothnia, Oulu, Finland

Sanna Siltanen Finnish Clinical Biobank Tampere / University of Tampere / Wellbeing Services County of Pirkanmaa, Tampere, Finland

Veli-Matti Kosma Biobank of Eastern Finland / University of Eastern Finland / Wellbeing services county of North Savo, Kuopio, Finland

Tiina Jokela Central Finland Biobank / University of Jyväskylä / Wellbeing Services County of Central Finland, Jyväskylä, Finland

**FinnGen Teams**

**Administration**

Anu Jalanko Institute for Molecular Medicine Finland (FIMM), HiLIFE, University of Helsinki, Helsinki, Finland

Risto Kajanne Institute for Molecular Medicine Finland (FIMM), HiLIFE, University of Helsinki, Helsinki, Finland

Mervi Aavikko Institute for Molecular Medicine Finland (FIMM), HiLIFE, University of Helsinki, Helsinki, Finland

Helen Cooper Institute for Molecular Medicine Finland (FIMM), HiLIFE, University of Helsinki, Helsinki, Finland

Denise Öller Institute for Molecular Medicine Finland (FIMM), HiLIFE, University of Helsinki, Helsinki, Finland

Tarja Laitinen Institute for Molecular Medicine Finland (FIMM), HiLIFE, University of Helsinki, Helsinki, Finland

Sofia Kuitunen University of Helsinki, Helsinki, Finland

**Sample and data logistics**

Auli Toivola Institute for Molecular Medicine Finland (FIMM), HiLIFE, University of Helsinki, Helsinki, Finland

Rodos Rodosthenous Institute for Molecular Medicine Finland (FIMM), HiLIFE, University of Helsinki, Helsinki, Finland

**Analysis**

Mitja Kurki Institute for Molecular Medicine Finland (FIMM), HiLIFE, University of Helsinki, Helsinki, Finland; Broad Institute, Cambridge, MA, United States

Juha Karjalainen Institute for Molecular Medicine Finland (FIMM), HiLIFE, University of Helsinki, Helsinki, Finland

Pietro Della Briotta Parolo Institute for Molecular Medicine Finland (FIMM), HiLIFE, University of Helsinki, Helsinki, Finland

Arto Lehisto Institute for Molecular Medicine Finland (FIMM), HiLIFE, University of Helsinki, Helsinki, Finland

Juha Mehtonen Institute for Molecular Medicine Finland (FIMM), HiLIFE, University of Helsinki, Helsinki, Finland

Reza Jabal Institute for Molecular Medicine Finland (FIMM), HiLIFE, University of Helsinki, Helsinki, Finland; Broad Institute, Cambridge, MA, United States

Mutaamba Maasha Institute for Molecular Medicine Finland (FIMM), HiLIFE, University of Helsinki, Helsinki, Finland; Broad Institute, Cambridge, MA, United States

Sanni Ruotsalainen Institute for Molecular Medicine Finland (FIMM), HiLIFE, University of Helsinki, Helsinki, Finland

Samuel Jones Institute for Molecular Medicine Finland (FIMM), HiLIFE, University of Helsinki, Helsinki, Finland

Raymond Walters Institute for Molecular Medicine Finland (FIMM), HiLIFE, University of Helsinki, Helsinki, Finland; Broad Institute, Cambridge, MA, United States

Paavo Häppölä Institute for Molecular Medicine Finland (FIMM), HiLIFE, University of Helsinki, Helsinki, Finland

**Disease Task Forces**

L. Elisa Lahtela Institute for Molecular Medicine Finland (FIMM), HiLIFE, University of Helsinki, Helsinki, Finland

Johanna Paltta Institute for Molecular Medicine Finland (FIMM), HiLIFE, University of Helsinki, Helsinki, Finland; University of Turku, Turku, Finland

Juulia Partanen Institute for Molecular Medicine Finland, HiLIFE, University of Helsinki, Finland

**Communication**

Mari Kaunisto Institute for Molecular Medicine Finland (FIMM), HiLIFE, University of Helsinki, Helsinki, Finland

**Sandbox & Cloud Services**

Elina Kilpeläinen Institute for Molecular Medicine Finland (FIMM), HiLIFE, University of Helsinki, Helsinki, Finland

Tianduanyi Wang Institute for Molecular Medicine Finland (FIMM), HiLIFE, University of Helsinki, Helsinki, Finland

Timo P. Sipilä Institute for Molecular Medicine Finland (FIMM), HiLIFE, University of Helsinki, Helsinki, Finland

Oluwaseun Alexander Dada Institute for Molecular Medicine Finland (FIMM), HiLIFE, University of Helsinki, Helsinki, Finland

Awaisa Ghazal Institute for Molecular Medicine Finland (FIMM), HiLIFE, University of Helsinki, Helsinki, Finland

Rigbe Weldatsadik Institute for Molecular Medicine Finland (FIMM), HiLIFE, University of Helsinki, Helsinki, Finland

Jaska Uimonen Institute for Molecular Medicine Finland (FIMM), HiLIFE, University of Helsinki, Helsinki, Finland

**Genotyping**

Kati Donner Institute for Molecular Medicine Finland (FIMM), HiLIFE, University of Helsinki, Helsinki, Finland

**Sample Collection Coordination**

Anu Loukola Helsinki Biobank / Helsinki University and Hospital District of Helsinki and Uusimaa, Helsinki

**Sample Logistics**

Päivi Laiho THL Biobank / Finnish Institute for Health and Welfare (THL), Helsinki, Finland

**Registry Data Operations**

Susanna Lemmelä Institute for Molecular Medicine Finland (FIMM), HiLIFE, University of Helsinki, Helsinki, Finland

Teemu Paajanen THL Biobank / Finnish Institute for Health and Welfare (THL), Helsinki, Finland

Arto Pietilä THL Biobank / Finnish Institute for Health and Welfare (THL), Helsinki, Finland

Aki Havulinna THL Biobank / Finnish Institute for Health and Welfare (THL), Helsinki, Finland

**Phenotype team**

Mary Pat Reeve Institute for Molecular Medicine Finland (FIMM), HiLIFE, University of Helsinki, Helsinki, Finland; Broad Institute, Cambridge, MA, United States

Shanmukha Sampath Padmanabhuni Institute for Molecular Medicine Finland (FIMM), HiLIFE, University of Helsinki, Helsinki, Finland

Harri Siirtola University of Tampere, Tampere, Finland

Javier Gracia-Tabuenca University of Tampere, Tampere, Finland

Marika Kaakinen Institute for Molecular Medicine Finland (FIMM), HiLIFE, University of Helsinki, Helsinki, Finland

Shuang Luo Institute for Molecular Medicine Finland (FIMM), HiLIFE, University of Helsinki, Helsinki, Finland

Vincent Llorens Institute for Molecular Medicine Finland (FIMM), HiLIFE, University of Helsinki, Helsinki, Finland

Dawit Yohannes Institute for Molecular Medicine Finland (FIMM), HiLIFE, University of Helsinki, Helsinki, Finland

**Data protection officer**

Iina Laak Institute for Molecular Medicine Finland (FIMM), HiLIFE, University of Helsinki, Helsinki, Finland

**FINBB – Finnish biobank cooperative**

Mervi Ahlroth Finnish Biobank Cooperative - FINBB

Johanna Mäkelä Finnish Biobank Cooperative - FINBB

Pauli Wihuri Finnish Biobank Cooperative - FINBB

Tom Southerington Finnish Biobank Cooperative - FINBB

Meri Lähteenmäki Finnish Biobank Cooperative – FINBB
